# Twin-based Mendelian Randomization Analyses Highlight Smoking’s Effects on Blood DNA Methylation, with Putative Reverse Causation

**DOI:** 10.1101/2024.06.19.24309184

**Published:** 2024-06-20

**Authors:** Madhurbain Singh, Conor V. Dolan, Dana M. Lapato, Jouke-Jan Hottenga, René Pool, Brad Verhulst, Dorret I. Boomsma, Charles E. Breeze, Eco J. C. de Geus, Gibran Hemani, Josine L. Min, Roseann E. Peterson, Hermine H. M. Maes, Jenny van Dongen, Michael C. Neale

**Affiliations:** 1. Virginia Institute for Psychiatric and Behavioral Genetics, Department of Psychiatry, Virginia Commonwealth University, Richmond, VA, USA; 2. Department of Human and Molecular Genetics, Virginia Commonwealth University, Richmond, VA, USA; 3. Department of Biological Psychology, Vrije Universiteit (VU) Amsterdam, Amsterdam, The Netherlands; 4. Amsterdam Public Health Research Institute, Amsterdam, The Netherlands; 5. Department of Psychiatry and Behavioral Sciences, Texas A&M University, College Station, TX, USA; 6. Division of Cancer Epidemiology and Genetics, National Cancer Institute, National Institutes of Health, Department Health and Human Services, Bethesda, MD, USA; 7. UCL Cancer Institute, University College London, London, UK.; 8. MRC Integrative Epidemiology Unit, University of Bristol, Bristol, UK; 9. Department of Psychiatry and Behavioral Sciences, SUNY Downstate Health Sciences University, Brooklyn, NY, USA; 10. Institute for Genomics in Health, SUNY Downstate Health Sciences University, Brooklyn, NY, USA; 11. These authors jointly supervised this work.; 12. Current address: Department of Complex Trait Genetics, Center for Neurogenomics and Cognitive Research, Vrije Universiteit (VU) Amsterdam, Amsterdam, The Netherlands

## Abstract

Epigenome-wide association studies (EWAS) aim to identify differentially methylated loci associated with complex traits and disorders. EWAS of cigarette smoking shows some of the most widespread DNA methylation (DNAm) associations in blood. However, traditional EWAS cannot differentiate between causation and confounding, leading to ambiguity in etiological interpretations. Here, we apply an integrated approach combining Mendelian Randomization and twin-based Direction-of-Causation analyses (MR-DoC) to examine causality underlying smoking-associated blood DNAm changes in the Netherlands Twin Register (N=2577). Evidence across models suggests that current smoking’s causal effects on DNAm likely drive many of the previous EWAS findings, implicating functional pathways relevant to several adverse health outcomes of smoking, including hemopoiesis, cell- and neuro-development, and immune regulation. Additionally, we find evidence of potential reverse causal influences at some DNAm sites, with 17 of these sites enriched for gene regulatory functional elements in the brain. The top three sites with evidence of DNAm’s effects on smoking annotate to genes involved in G protein-coupled receptor signaling (*GNG7*, *RGS3*) and innate immune response (*SLC15A4*), elucidating potential biological risk factors for smoking. This study highlights the utility of integrating genotypic and DNAm measures in twin cohorts to clarify the causal relationships between health behaviors and blood DNAm.

## Introduction

Epigenome-wide association studies (EWASs) are valuable for identifying variation in DNA methylation (DNAm) associated with complex human traits and diseases^1^. By far, the most successful EWASs have been the studies of cigarette smoking. A large-scale EWAS meta-analysis of smoking (N = 15,907 individuals) compared current versus never smoking to reveal significant DNAm differences at 18,760 CpG (*Cytosine-phosphate-Guanine*) sites in peripheral blood cells^2^. DNAm differences between former- and never-smoking individuals were diminished but remained statistically significant at 2,568 sites^2^. Genes annotated to the differentially methylated CpG sites have been implicated in genome-wide association studies (GWAS) of several smoking-associated traits, including cancers, lung functions, cardiovascular disorders, inflammatory disorders, and schizophrenia, indicating DNAm’s potential role in the adverse health effects of smoking^2^.

As cross-sectional EWAS in unrelated individuals cannot differentiate between causation and confounding^3^, the widespread associations between cigarette smoking and DNAm^2^ may originate from a combination of different etiological mechanisms. These associations are typically interpreted as the causal *effects* of smoking exposure on DNAm. However, some smoking-associated CpG sites may have reverse or bidirectional causal links with smoking, such that DNAm may reciprocally affect the development and maintenance of smoking behaviors^4^. Moreover, associations between smoking and DNAm could be attributable to potential confounders, such as schizophrenia^5^, alcohol consumption^6^, cannabis use^7^, and body mass index^8^.

An alternative approach to causal inference in observational studies is Mendelian Randomization (MR) analysis, using genetic variants as instrumental variables (IVs) to estimate causal effects under specific assumptions^3,9^ (see Methods). Previous MR analyses have identified potential effects of lifetime (current or former) smoking liability on blood DNAm at only 11 CpG sites^10^, along with potential reverse effects of blood DNAm at 9 sites^11^. The causal inference in MR is based on the assumption that the genetic variants associated with the exposure influence the outcome exclusively through the exposure. In other words, the genetic variants used as IVs for smoking may show vertical pleiotropy, but not horizontal pleiotropy, with DNAm. To minimize potential violations of these assumptions, MR analyses require carefully selected single-nucleotide polymorphisms (SNPs), including using genetic colocalization to filter out SNPs showing horizontal pleiotropy due to linkage disequilibrium (LD). Since individual SNPs usually have minuscule effect sizes on complex traits, traditional MR approaches using a few selected SNPs may have limited power to detect causality and may be subject to weak-instrument bias^12^.

Recent methodological developments^13,14^ integrate the principles of MR with the twin-based *Direction of Causation* model (hence called *MR-DoC*) from biometrical studies of mono- and dizygotic twins^15^. Causal inference in twin data leverages the cross-twin cross-trait correlations to estimate the direction and magnitude of potential causal effects between traits^18^. Further, the MR-DOC approaches, i.e., the unidirectional *MR-DoC1*^13^ and the bidirectional *MR-DoC2*^14^, help account for some of the horizontal pleiotropic associations of the genetic IV with the outcome, unmediated by the exposure trait. Consequently, MR-DoC models allow using polygenic risk scores (PRS) as potential IVs, increasing the statistical power to estimate causal effects while curtailing weak-instrument bias relative to traditional MR methods that use SNPs as IVs. Incorporating MR with family data also helps to resolve additional assumptions of standard MR, such as random mating and no dyadic effects^13,16^.

**Figure 1.**
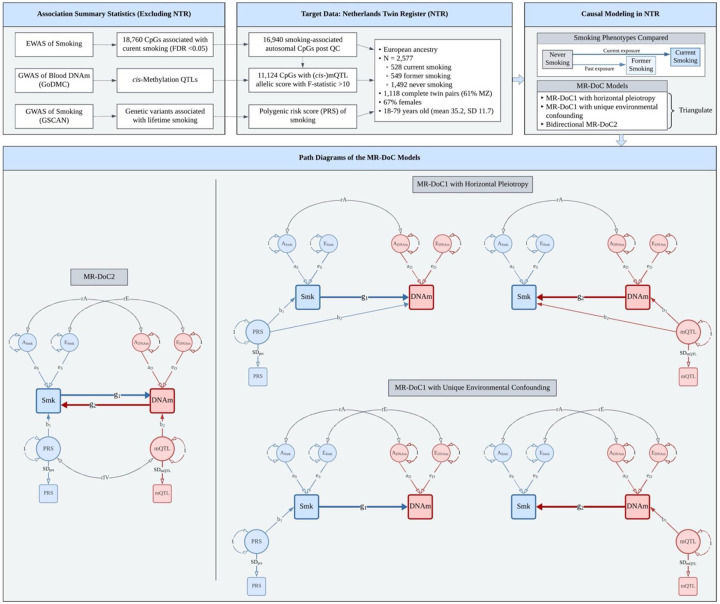
Study Design. Overview of the data and MR-DoC models used to examine the causality between cigarette smoking and blood DNA methylation (DNAm) in the Netherlands Twin Register. The models were fitted separately for current (versus never) and former (versus never) smoking. Applying the five MR-DoC models shown in the path diagrams, we obtained a set of three causal estimates in each direction of causation: Smoking (Smk) → DNAm (the blue paths labeled **g**_**1**_) and DNAm → Smoking (the red paths labeled **g2**). Note. For better readability, the path diagrams show only the within-individual part of the models fitted to data from twin pairs. The squares/rectangles indicate observed variables, the circles indicate latent (unobserved) variables, the single-headed arrows indicate regression paths, and the double-headed curved arrows indicate (co-)variances.

The present study used MR-DoC models to examine bidirectional causal effects between cigarette smoking and peripheral blood DNAm in European ancestry adult twins from the Netherlands Twin Register (NTR)^17^ (Figure 1). The target sample included 2,577 individuals from 1,459 twin pairs with both genotypic and DNAm data, as well as their self-reported smoking status at the time of blood draw (comprising 528 currently, 549 formerly, and 1,492 never-smoking individuals). Across 16,940 smoking-related CpGs previously identified^2^, we fitted separate models for current (versus never) and former (versus never) smoking. We obtained a set of three causal estimates in each direction (*Smoking → DNAm*, and *DNAm → Smoking*): the estimates from bidirectional MR-DoC2 and two different model specifications of unidirectional MR-DoC1 (Figure 1). We triangulated evidence across the three models based on the statistical significance and consistency of the causal estimates. The results indicated more widespread putative causal influences of smoking on DNAm than *vice versa*. Follow-up enrichment analyses highlighted biological processes and tissues relevant to the CpG sites with potential effects in either direction of causation.

## Results

### *cis*-mQTLs identified for two-thirds of smoking-associated CpG sites

**Figure 2.**
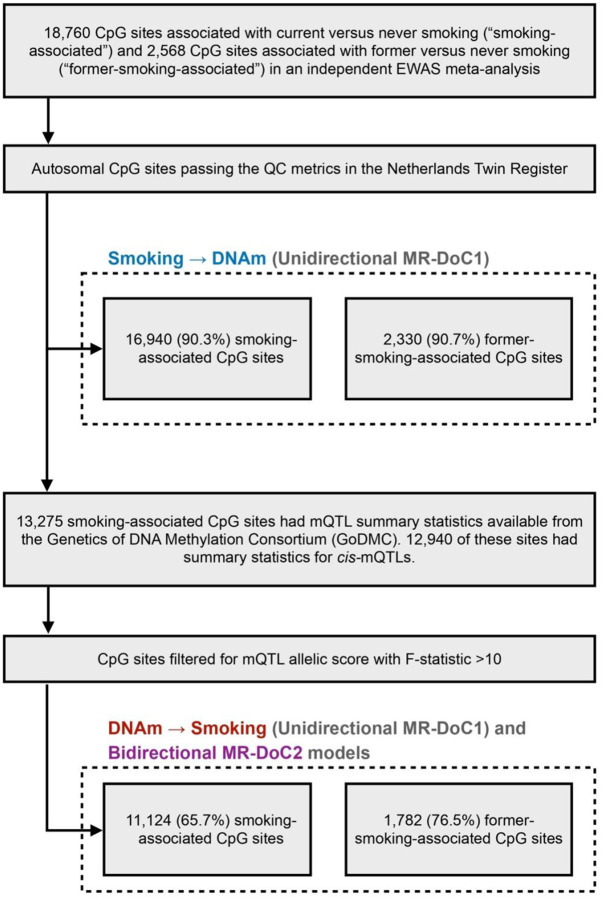
Selection of CpG sites tested in each MR-DoC model. Previous independent EWAS meta-analysis of cigarette smoking^2^ examined DNA methylation (DNAm) at CpG sites from the Illumina HumanMethylation450 BeadChip array^21^, which was also used to measure DNAm in the NTR biobank. In the unidirectional MR-DoC1 models for Smoking → DNAm, we included autosomal CpG sites associated with smoking in the EWAS meta-analysis that also passed the QC metrics in NTR. The MR-DoC1 models for DNAm → Smoking and the bidirectional MR-DoC2 models were restricted to a subset of these sites having cis-mQTL summary statistics from the GoDMC^18^ and a resulting mQTL allelic score with F-statistic >10.

We used a weighted sum of relevant DNAm-increasing alleles at *cis*-methylation quantitative trait loci (henceforth called *mQTL allelic score*) as the IV for DNAm. Of the 18,760 CpG sites associated with current smoking in a previous independent EWAS meta-analysis^2^, 16,940 autosomal sites passed the QC metrics in NTR (hereafter called the “smoking-associated CpGs”) and were analyzed in the unidirectional MR-DoC1 models for *Current Smoking → DNAm* (Figure 2). Of these sites, 13,275 had mQTL summary statistics from the Genetics of DNA Methylation Consortium (GoDMC; excluding NTR)^18^. A subset of 12,940 sites had summary statistics for *cis*-mQTLs, i.e., SNPs within 1Mb of the CpG. We used only *cis*-mQTLs to derive the IVs for DNAm, given that SNPs located close to the CpG are more likely to be associated with smoking *via* DNAm. To further guard against potential horizontal pleiotropy with smoking, we relied on the consistency of the causal estimates in MR-DoC models accommodating horizontal pleiotropy. To reduce the risk of weak-instrument bias in the estimated effects of DNAm on smoking, we restricted the MR-DoC1 models for *DNAm → Current Smoking* and the bidirectional MR-DoC2 models to 11,124 (65.7%) smoking-associated CpGs having an mQTL allelic score with F-statistic >10, the criterion for the “relevance” assumption of a valid IV^19^ (see Methods). The included mQTL allelic scores had an incremental R^2^ for the respective CpG site ranging from 0.43% to 76.95% (mean 9.04%, S.D. = 10.94%). Applying similar inclusion criteria, we identified 2,330 autosomal, post-QC CpG sites previously associated with former smoking^2^ (hereafter called the “former-smoking-associated CpGs”), which were analyzed in the MR-DoC1 models for *Former Smoking → DNAm*. A subset of 1,782 (76.5%) former-smoking-associated CpGs had mQTL allelic scores with F-statistic >10 and were examined in the MR-DoC1 models for *DNAm → Former Smoking* and the bidirectional models.

We used a PRS of lifetime regular-smoking initiation^20^ as the IV for smoking status, which had an incremental liability-scale R^2^ of 5.07% (F-statistic = 73.2) for current versus never smoking and 2.02% (F-statistic = 28.8) for former versus never smoking in the target NTR dataset.

### Exemplar: Putative causality between current smoking and *AHRR* DNAm

To illustrate the three MR-DoC models, we first present the results for two CpG sites (cg23916896 and cg05575921) in the Aryl-Hydrocarbon Receptor Repressor (*AHRR*) gene, which are among the most well-established DNAm signatures of cigarette smoking^2^.

One of the two MR-DoC1 model specifications allowed us to estimate and account for potential unbalanced horizontal pleiotropy from the mQTL allelic score to smoking in *DNAm → Smoking* models and from the smoking PRS to DNAm in *Smoking → DNAm* models. However, to estimate this pleiotropic association, the model requires fixing the confounding due to unique environmental factors to a specific value (here, zero)^13^. In the second specification of MR-DoC1, we freely estimated and controlled for potential unique environmental confounding (labeled “rE” in Figure 1), while instead assuming that the IV had no horizontal pleiotropy. In MR-DoC2 models, we estimated bidirectional causal effects by including both the smoking PRS and the mQTL allelic score, while allowing the two IVs to covary with each other^14^. Covariance between the PRS and the mQTL allelic score may arise from many possible sources, including shared pleiotropic SNPs, LD between the constituent SNPs, and population structure. Therefore, MR-DoC2 may help reduce potential biases in the causal estimates by accounting for these sources of covariance between smoking PRS and mQTL allelic score. Across all models, causal relationships with the binary smoking variable are estimated on the latent liability scale^22^. So, even where smoking is the “exposure” variable, the causal estimate is interpreted as the effect of the underlying smoking *liability* rather than smoking *exposure*.

**Figure 3.**
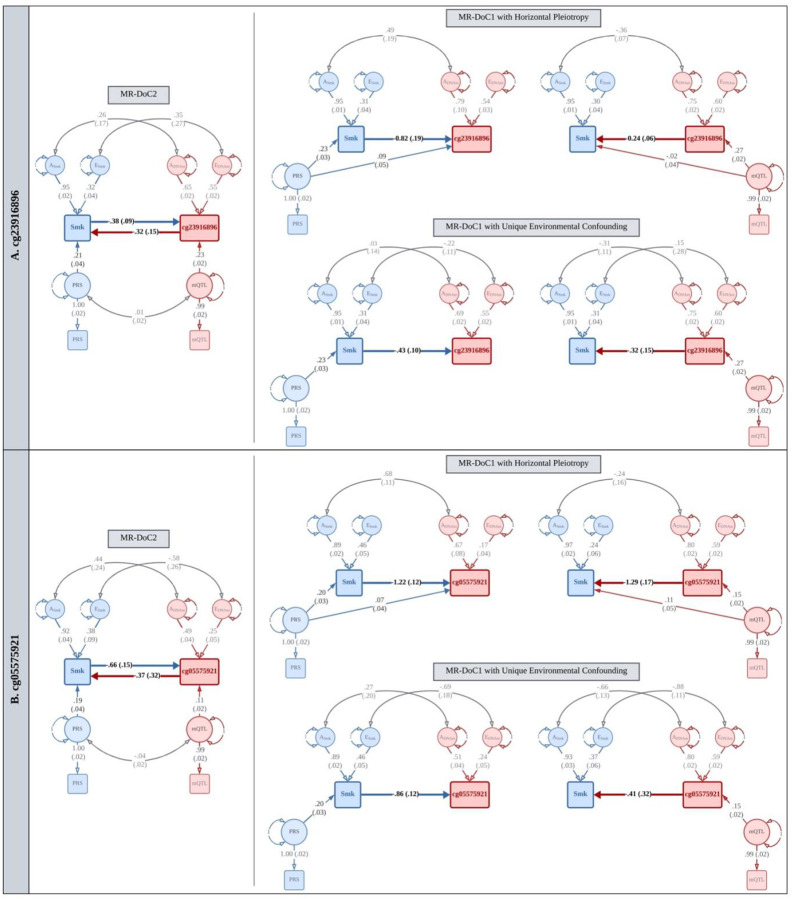
Illustrative MR-DoC models of causality between current smoking and blood DNAm at (A) cg23916896 and (B) cg05575921 in the AHRR gene. We fitted five MR-DoC models at each CpG: (1) Smoking → DNAm MR-DoC1 with horizontal pleiotropy, (2) Smoking → DNAm MR-DoC1 with unique environmental confounding, (3) DNAm → Smoking MR-DoC1 with horizontal pleiotropy, (4) DNAm → Smoking MR-DoC1 with unique environmental confounding, and (5) bidirectional MR-DoC2. Thus, for each CpG, three causal estimates were obtained in either direction of causation. In the path diagrams, squares/rectangles indicate observed variables, circles indicate latent (unobserved variables), single-headed arrows indicate regression paths, and double-headed curved arrows indicate (co-)variance. The residual variance of smoking status liability is partitioned into additive genetic (A_Smk_) and unique environmental (E_Smk_) components. Likewise, the residual variance of DNAm is partitioned into A_DNAm_ and E_DNAm_. The correlation between A_Smk_ and A_DNAm_ represents the confounding between smoking and DNAm due to latent (unobserved) additive genetic factors, while the correlation between E_Smk_ and E_DNAm_ represents confounding due to latent unique environmental factors. Each model included age and sex as covariates of smoking status (not shown). DNAm β-values were residualized for standard biological and technical covariates used in EWAS (see Methods). The smoking PRS and the mQTL allelic scores were residualized for standard GWAS covariates, including genetic principal components and genotyping platform. In the path diagrams, the residualized PRS and mQTL allelic scores are regressed on respective latent factors, representing the underlying “true” standardized scores (mean = zero; variance = one). The coefficient of the path from the latent score to the observed score estimates the standard deviation of the observed score. Note. The paths are labeled by the point estimate and its S.E. in parentheses. For better readability, the path diagrams show only the within-individual part of the models fitted to data from twin pairs.

For probe cg23916896 (Figure 3A), the mQTL allelic score had an incremental R^2^ of 8.03% (F-statistic = 156.4). The estimated effects indicated that higher liability for current smoking likely causes hypomethylation of cg23916896, with consistently negative causal estimates: −0.82 (95% confidence interval: −1.20, −0.44) in MR-DoC1 with horizontal pleiotropy, −0.43 (−0.62, −0.24) in MR-DoC1 with unique environmental confounding, and −0.38 (−0.55, −0.21) in the bidirectional MR-DoC2 model. These estimates remained statistically significant after FDR correction in all three models. The estimated reverse effect of cg23916896 methylation on the liability for current smoking also had consistently negative estimates in all models: −0.24 (−0.37, −0.12) in MR-DoC1 with horizontal pleiotropy, −0.32 (−0.61, −0.04) in MR-DoC1 with unique environmental confounding, and −0.32 (−0.61, −0.04) in MR-DoC2. That is, hypomethylation of cg23916896 putatively increases the liability for current smoking. These estimates were statistically significant at false discovery rate (FDR) <0.05 in MR-DoC1 with horizontal pleiotropy, but only nominally significant (p <0.05) in the other two models. Taken together, these results provide robust evidence for current smoking’s causal effects on cg23916896 methylation, with suggestive evidence for the reverse effect of cg23916896 methylation on smoking. Previous MR studies of lifetime smoking and DNAm have not examined this CpG site, as these studies focused on a few selected sites^10,11^. Our analyses indicate that cg23916896 potentially has a bidirectional causal relationship with cigarette smoking, such that the smoking-induced hypomethylation at this locus may reciprocally increase the liability for smoking.

In comparison, probe cg05575921 (one of the CpGs most robustly associated with cigarette smoking) had an mQTL allelic score with a relatively modest incremental R^2^ of 1.74% (F-statistic = 31.6). Similar to cg23916896, the effect of current smoking liability on cg05575921 methylation had consistently negative, robust estimates, with FDR <0.05 in all three models (Figure 3B), which also aligns with the previously reported negative, albeit non-significant, effect of *lifetime* smoking^10^. The reverse effect of cg05575921 methylation on smoking liability was estimated to be −1.29 (−1.62, −0.96) in MR-DoC1 with horizontal pleiotropy, −0.41 (−1.03, 0.21) in MR-DoC1 with unique environmental confounding, and −0.37 (−1.00, 0.26) in MR-DoC2. Although the point estimates were negative in all three models, they were not statistically significant in the latter two models. Notably, the point estimates for cg05575921 are comparable to those for cg23916896 but have larger standard errors, likely due to the former’s weaker IV (mQTL allelic score).

### Evidence of more widespread effects of current smoking on DNAm than *vice versa*

To evaluate whether there was evidence of widespread, small causal effects of current smoking on DNAm, we examined the Bayesian genomic inflation factor^23^ (λ) using p-values of the causal estimates. Across the 16,940 smoking-associated CpG sites, MR-DoC1 with horizontal pleiotropy had λ = 1.44, while MR-DoC1 with unique environmental confounding showed λ = 1.20. For comparison, fitting similar models epigenome-wide at 411,169 autosomal, post-QC CpGs showed much less inflation (λ = 0.98 and λ = 1.09, respectively), suggesting enrichment of low p-values among the smoking-associated CpGs. The epigenome-wide inflation is in line with that for cigarettes per day (λ >1.1) previously reported using two-sample MR^18^. Corresponding QQ plots showed a deviation of the causal estimate p-values from the null hypothesis across a broad range of smoking-associated CpG sites (Supplementary Figures S1, S2). Across the 11,124 CpG sites with bidirectional MR-DoC2 models, the estimated reverse effects of DNAm on current smoking showed little inflation (λ = 1.01) compared to the effects of current smoking on DNAm in the same model (λ = 1.20; Supplementary Figures S3, S4). These findings suggest that the causal influences of current smoking on DNAm likely contribute, at least partly, to the previously reported EWAS hits. For the reverse effects of DNAm on current smoking, the absence of λ inflation does not preclude potential localized small effects at several CpG sites. Furthermore, despite the inflation of the test statistics, our sample size might be insufficient to obtain significant estimates of relatively small effects in either direction of causation.

**Figure 4.**
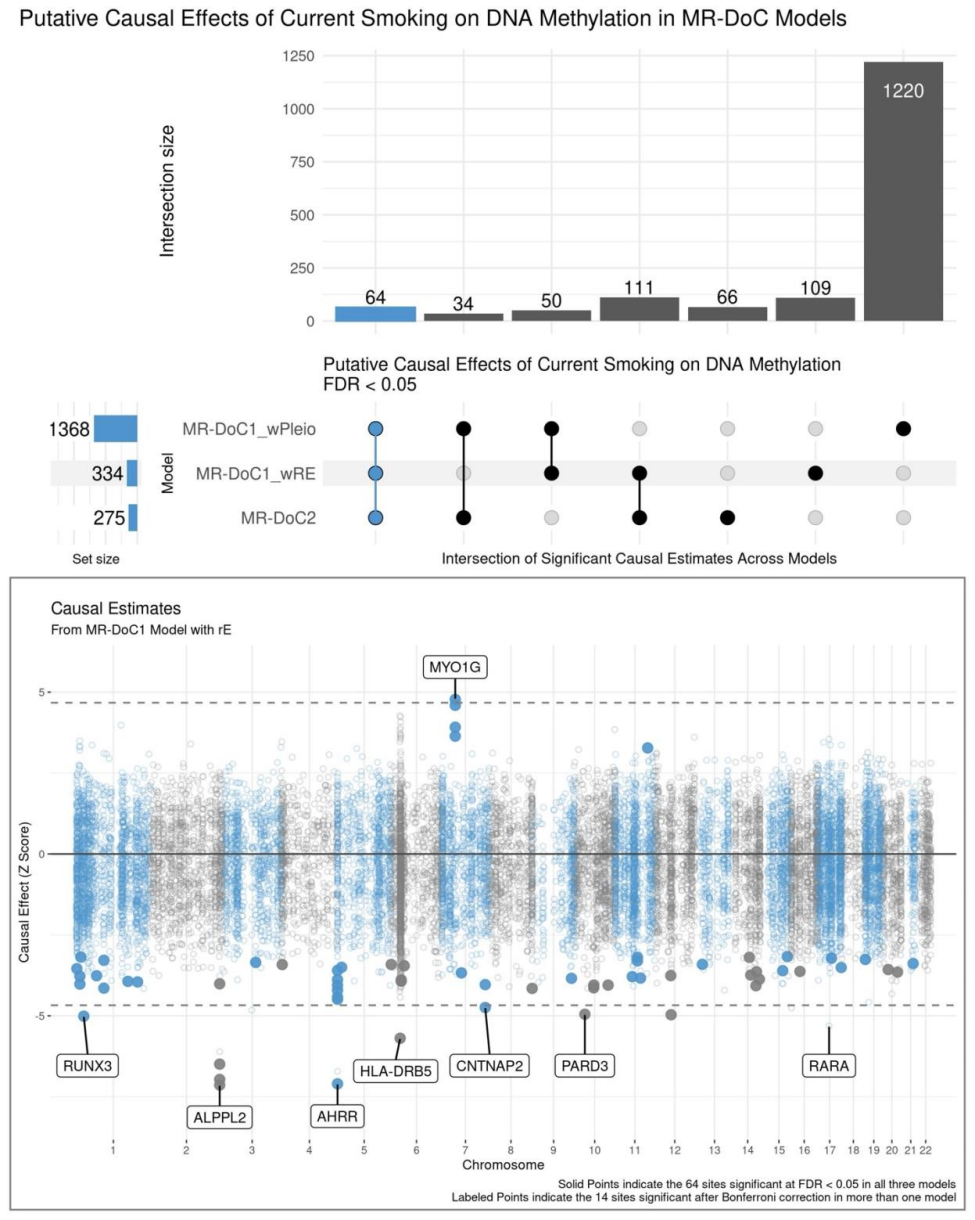
Putative Causal Effects of Current Smoking on Blood DNA Methylation in MR-DoC Models The top panel shows an UpSet plot of the intersection of CpG sites with statistically significant (FDR <0.05) estimates of Current Smoking → DNAm in the three MR-DoC models. The matrix consists of the models along the three rows and their intersections along the columns. The horizontal bars on the left represent the number of CpGs with significant (FDR <0.05) causal estimates in each model. The vertical bars represent the number of CpGs belonging to the respective intersection in the matrix. The bottom panel shows a Miami plot of the Current Smoking → DNAm causal estimates across 16,940 smoking-associated CpGs. The X-axis shows the genomic positions of the CpG sites aligned to Genome Reference Consortium Human Build 37 (GRCh37). The Y-axis shows the Z-statistic of the estimated effect of the liability for current (versus never) smoking on (residualized and standardized) DNA methylation β-values in the MR-DoC1 model with unique environmental confounding (rE). The solid points indicate the 64 sites with significant causal estimates (FDR <0.05) in all three models (i.e., the blue vertical bar in the UpSet plot). The CpG sites with causal estimates significant after Bonferroni correction in more than one model are labeled by their respective nearest gene. Note. The data underlying these plots are in Supplementary Table S1.

There also was considerable variability in the number of CpG sites with statistically significant causal estimates across models. The estimated *Current Smoking → DNAm* effects had FDR <0.05 at 1,368 CpGs in MR-DoC1 with horizontal pleiotropy, 334 CpGs in MR-DoC1 with unique environmental confounding, and 275 CpGs in MR-DoC2 (Figure 4; **top panel**). The relatively higher number of statistically significant causal estimates in MR-DoC1 with horizontal pleiotropy may partly be due to its higher power compared to the other models^24^. Looking at the intersection of significant estimates across models, 259 CpG sites showed FDR <0.05 in at least two models, while 64 sites showed FDR <0.05 in all three models. These 64 sites also showed consistency in the direction of effect across all three models (Supplementary Figure S5, Table S1). Thus, we considered these 64 CpG sites to exhibit robust evidence for the causal effects of current smoking liability on DNAm, including hypomethylation of 59 sites and hypermethylation of the other five (Figure 4; **bottom panel**). These CpGs are annotated to some of the top genes implicated in prior EWAS of smoking^2^, including hypomethylation of CpGs in/near *AHRR*, *ALPPL2* (alkaline phosphatase placental-like 2), *CNTNAP2* (contactin-associated protein 2), and *PARD3* (par-3 family cell polarity regulator) and hypermethylation of CpGs in *MYO1G* (myosin 1G). Only one of these 64 CpG sites lies within the major histocompatibility complex (MHC) region (chr6:28477797–33448354): cg06126421 located near gene *HLA-DRB5*. Due to its complex LD structure, the causal estimates of the sites in the MHC region should be interpreted with caution.

On applying a more conservative Bonferroni correction for multiple testing, 14 sites had significant *Current Smoking → DNAm* causal estimates in more than one model, while only four CpGs had significant estimates in all three models (Supplementary Figure S6). Thus, these four CpGs showed the most robust evidence for the effects of current smoking on DNAm, comprising three sites with hypomethylation (cg05951221 and cg01940273 near *ALPPL2*, and cg06126421 near *HLA*-*DRB5*) and one with hypermethylation (cg12803068 in *MYO1G*).

**Figure 5.**
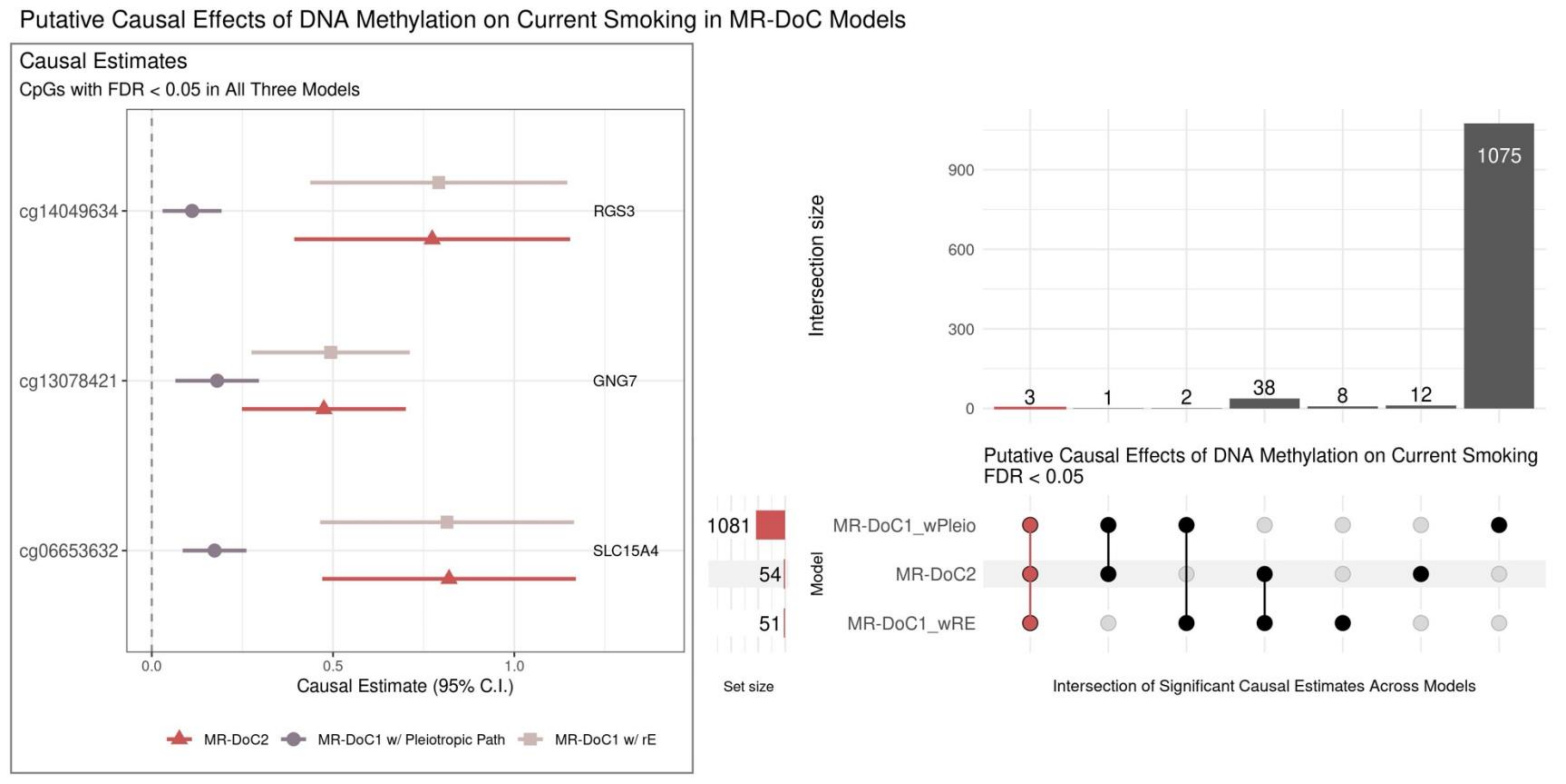
Putative causal effects of blood DNA methylation on current-smoking liability in MR-DoC models The left panel shows the estimates and Wald-type 95% confidence intervals of the causal effects of (residualized and standardized) DNA methylation β-values on the liability for current (versus never) smoking in each of the three MR-DoC models: bidirectional MR-DoC2, MR-DoC1 with horizontal pleiotropic path, and MR-DoC1 with unique environmental confounding (rE). The text labels indicate the gene to which the CpG is annotated. The right panel shows an UpSet plot of the intersection of CpG sites with statistically significant (FDR <0.05) estimates of DNAm → Current Smoking in each of the three MR-DoC models. The matrix consists of the models along the three rows and their intersections along the columns. The horizontal bars on the left represent the number of CpGs with significant (FDR <0.05) causal estimates in each model. The vertical bars represent the number of CpGs belonging to the respective intersection in the matrix. Note. The data underlying these plots are in Supplementary Table S3.

The estimated *DNAm → Current Smoking* effects were significant (FDR <0.05) at 1,081 CpGs in MR-DoC1 with horizontal pleiotropy, 51 CpGs in MR-DoC1 with unique environmental confounding, and 54 CpGs in MR-DoC2 (Figure 5; **right panel**). Further, 44 CpGs showed FDR <0.05 in at least two models, but only three CpGs had FDR <0.05 in all three models. The three CpGs also had consistent, positive estimates across models, suggesting that hypermethylation of CpG sites in *GNG7* (G-Protein Subunit Gamma 7), *RGS3* (Regulator of G-Protein Signaling 3), and *SLC15A4* (Solute Carrier Family 15 Member 4) genes may increase the liability for current smoking (Figure 5; **left panel**). None of these sites has been previously reported to have effects on smoking liability^11^. Applying the more conservative Bonferroni correction, nine CpGs had significant *DNAm → Current Smoking* causal estimates in more than one model, but none showed Bonferroni-corrected significant effects in all three models (Supplementary Figure S7).

### Suggestive Evidence of Bidirectional Effects at Four CpG Sites

**Figure 6.**
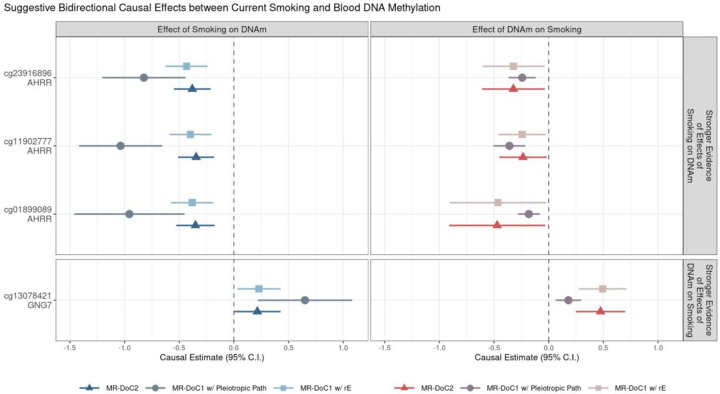
Potential bidirectional effects between current smoking and blood DNA methylation Estimates and Wald-type 95% confidence intervals of bidirectional causal effects between the liability for current (versus never) smoking and (residualized and standardized) DNA methylation β-values in the three MR-DoC models: bidirectional MR-DoC2, MR-DoC1 with horizontal pleiotropic path, and MR-DoC1 with unique environmental confounding (rE). The Y-axis labels indicate the CpG probe IDs and the respective genes in which the CpGs are located. Three of the four CpGs are in the AHRR gene and show robust evidence of the causal effects of current smoking on DNAm, along with weaker evidence of the reverse effects of DNAm on smoking. On the other hand, the fourth CpG is located in the GNG7 gene and shows robust evidence of the causal effects of DNAm on current smoking, with weaker evidence of the reverse effects of smoking on DNAm. Note. The data underlying these plots are in Supplementary Tables S1–S4.

The 64 CpG sites with robust evidence of current smoking’s effects on DNAm do not overlap with the three sites with robust evidence of reverse effects. However, further examining the causal estimates revealed that three of the 64 sites also had consistently negative, nominally significant (p <0.05) estimates of *DNAm → Current Smoking* effects in all models (Figure 6). The three CpGs (cg23916896, cg11902777, cg01899089) are all located in the *AHRR* gene, suggesting potential bidirectional effects between current smoking and *AHRR* DNAm. That is, current smoking putatively causes hypomethylation of CpGs in *AHRR*, which, in turn, may further increase smoking liability as a feedback effect. Among the CpGs with robust evidence of DNAm’s effects on current smoking, cg13078421 (*GNG7*) also showed consistently positive, nominally significant estimates of current smoking’s effects on DNAm. Thus, *GNG7* hypermethylation putatively increases smoking liability, with a potential reverse effect of current smoking on *GNG7* methylation. Additionally, 15 CpGs had consistent, nominally significant bidirectional causal estimates in all three models, though the estimates were not significant after FDR correction in either direction (Supplementary Figure S8).

### DNAm loci potentially influenced by smoking are enriched for biological processes relevant to smoking’s adverse health outcomes

For follow-up gene-set annotation and functional enrichment analyses^25^, we identified 525 CpG sites (outside the MHC region) with potential effects of current smoking on DNAm based on consistent, nominally significant estimates in all three models (Supplementary Table S1). The genes mapped by these CpGs showed extensive significant enrichment (FDR <0.05) for ontology clusters, including hemopoiesis, cell morphogenesis, inflammatory response, regulation of cell differentiation, and regulation of nervous system development, underscoring DNAm’s potential role in the adverse health sequelae of smoking (Supplementary Figures S9–S11; Tables S5–S6).

Next, we performed *eFORGE 2.0* (experimentally derived Functional element Overlap analysis of ReGions from EWAS)^26,27^ analyses to explore the tissue-specific functional relevance of these CpG sites. These sites were significantly enriched (FDR <0.05) for overlap with a wide range of gene regulatory elements, including chromatin states, histone marks, and DNase-I hotspots, in most of the tissue/cell types in reference datasets. These findings suggest that the functional consequences of the effects of smoking on DNAm are likely widespread across the body rather than specific to a few tissue types (Supplementary Figures S12–S14; Tables S7–S9).

### CpG sites with consistent effects on current smoking show enrichment for brain-related gene regulatory elements

For potential *DNAm → Current Smoking* effects, we identified 64 CpGs with consistent, nominally significant estimates in all three models (Supplementary Figure S15). In the gene-set enrichment analyses (Supplementary Figures S16–S17; Tables S10–S11), the genes mapped by these CpGs did not show significant functional enrichment (FDR <0.05), likely due to too few loci implicated in this direction of causation. However, in the eFORGE analyses, which use precise chromatin-based information for each CpG, these CpG sites showed significant enrichment (FDR <0.05) for overlap with enhancers in the brain (fetal brain), blood (primary B cells, hematopoietic stem cells), lung, and mesodermal embryonic stem cells (Supplementary Figures S18–S20; Tables S12–S14). This set of CpGs also showed significant enrichment for histone marks in multiple tissues/cell types (including the brain, blood, and lung), but the overlap with DNase-I hotspots was not significantly enriched. The tissues/cell types predicted to be relevant for DNAm’s effects on smoking liability may be prioritized for follow-up tissue-/cell type-specific studies.

**Figure 7.**
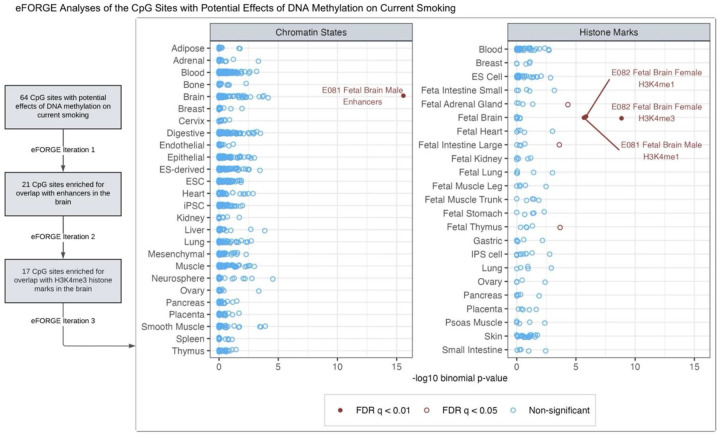
Among the CpG sites with potential effects of blood DNA methylation on current smoking liability, iterative eFORGE analyses elucidated sites enriched for overlap with brain-related chromatin states and histone marks. The first iteration of eFORGE examined the 64 CpG sites with potential effects of blood DNA methylation on current smoking liability (Supplementary Figure S15), revealing 21 CpGs enriched for overlap with enhancers in the brain (Supplementary Figure S18/Table S12). In follow-up analyses restricted to these 21 CpGs (eFORGE iteration 2), all 21 probes were also enriched for the brain H3K4me1 marks, while 17 of these probes overlapped with H3K4me3 marks in the brain (Supplementary Figure S22/Table S16). This iteration also showed significant enrichment (FDR q <0.01) for histone marks in other tissues, including small and large intestines, adrenal gland, and thymus. So, to identify a subset of these CpGs with potentially more specific enrichment for brain-related functional elements, we restricted further analyses to the 17 sites overlapping with the brain H3K4me3 marks (eFORGE iteration 3). As seen in this figure, these 17 sites showed highly specific enrichment for enhancers and histone marks in the brain (Supplementary Tables S18–S19). Ten of these sites also overlapped with DNase-I hotspots in the brain (Supplementary Table S20).

To further gauge the tissue-specificity of the eFORGE enrichment, we performed iterative follow-up analyses with the CpGs overlapping with tissue/cell types of interest (see Methods and Supplementary Figures S21–S23; Tables S15–S17). These analyses elucidated a subset of 17 CpGs with significant and highly specific enrichment for enhancers and histone marks (H3K4me1 and H3K4me4) in the brain (Figure 7), along with weaker enrichment for H3K4me1 in the adrenal gland and thymus. Ten of the 17 sites also overlapped with DNase-I hotspots in the brain, though the enrichment was not statistically significant (FDR = 0.08) (Supplementary Figure S24, Table S20). The causal estimates and the nearest gene of these 17 CpGs are shown in Supplementary Figure S25. Four of these CpGs also had consistent estimates of the reverse effects of current smoking on DNAm (identified by the column “g1_nominal” in Supplementary Table S4): cg25612391 (*SLC25A42*), cg05424060 (*GNAI1*), cg10590964 (near *KIAA2012*), and cg05877788 (*TP53I13*). Furthermore, prior pre-clinical and clinical studies have implicated 14 of the 17 mapped genes, including three with potential bidirectional effects, in behavioral or neurological traits, such as alcohol dependence (*OSBPL5*)^28^, cocaine use (*SLCO5A1*)^29^, anxiety (*CCDC92*)^30^, depression (*GNAI1*)^31^, encephalomyopathy and brain stress response (*SLC25A42*)^32,33^, and dementia or Alzheimer’s disease pathology (*SIAH3*, *SRM*, *TP53I13*)^34–36^.

Similar follow-up analyses with other subsets of CpGs (e.g., probe sets enriched for enhancers in the lung or cord blood primary B cells) showed enrichment across several tissue/cell types, suggesting non-specificity of the enrichment seen in these tissues (Supplementary Figures S26–S31; Tables S21–S26). The enrichment for specific blood cell types (here, B cells) may be partly confounded by residual cell-composition effects in whole blood analyses^26^. The 18 CpGs overlapping with enhancers in primary B cells mapped to 16 genes, of which five have been previously associated with (any) blood cell counts but only one with lymphocyte count in GWAS^37^. Thus, the sites driving the enrichment for B cells had little overlap with the known lymphocyte-count GWAS associations. For comparison, the 64 CpGs with potential *DNAm → Current Smoking* effects annotated to 51 genes, of which 16 are known to be associated with (any) blood cell counts and only two with lymphocyte count.

### Attenuated effects of former smoking on DNAm

MR-DoC analyses estimating the causal effects between former smoking and DNAm showed attenuated inflation factor (λ) in all models, compared to the λ values in similar models fitted to current smoking. For instance, the MR-DoC2 models fitted across the 11,124 smoking-associated CpGs had λ = 1.11 for *Former Smoking → DNAm* and λ = 0.99 for *DNAm → Former Smoking*, compared to 1.20 and 1.01, respectively, for current smoking. Note that these λ calculations were not restricted to the former-smoking-associated CpGs to allow for a comparison with current smoking.

**Figure 8.**
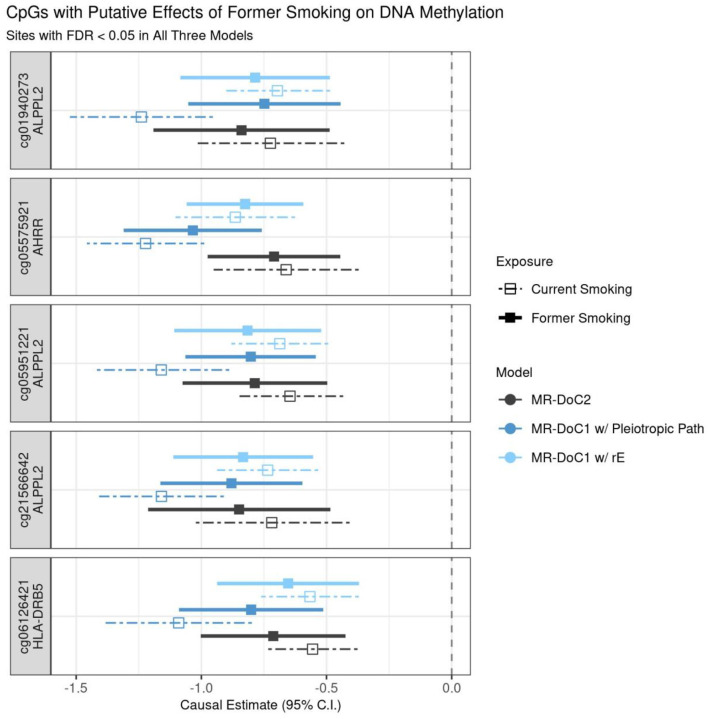
Putative causal effects of former smoking on blood DNA methylation. Estimates and Wald-type 95% confidence intervals of the causal effects of the liability for former (versus never) smoking and (residualized and standardized) DNA methylation beta-values in each of the three MR-DoC models: bidirectional MR-DoC2, MR-DoC1 with horizontal pleiotropic path, and MR-DoC1 with unique environmental confounding (rE). The corresponding estimates for current (versus never) smoking are also shown with dashed lines. The text labels on the left indicate the CpG probe IDs and the genes mapped by the CpGs. Note. The data underlying these plots are in Supplementary Tables S1 and S27, indicated by the column g1_robust.

Among the former-smoking-associated CpGs, only five sites showed robust evidence of causal effects of former smoking on DNAm, with consistent, statistically significant (FDR <0.05) causal estimates in all three models (Supplementary Figure S32). These CpGs include cg05575921 in *AHRR*, cg05951221, cg01940273, and cg21566642 near *ALPPL2*, and cg06126421 near *HLA-DRB5* gene (in the MHC region). The causal estimates at these sites are similar to those of the effects of current smoking on DNAm, with overlapping confidence intervals (Figure 8). Thus, unlike most smoking-associated CpGs^38^, smoking’s effects on DNAm at these sites likely have limited reversibility, in line with the previously reported persistent associations of these sites with former smoking 30 years after cessation^2^. For the reverse effects of DNAm on former smoking, no CpG showed consistent (at least nominally significant) causal estimates across models (Supplementary Figure S33). Nevertheless, of the three CpGs with robust evidence of DNAm’s effects on current smoking, two were among the former-smoking-associated CpGs and had overlapping confidence intervals of the estimated effects of DNAm on *former* smoking and *current* smoking (Supplementary Figure S34).

## Discussion

Results from integrated MR and biometrical genetic (MR-DoC) modeling suggest that the causal effects of cigarette smoking on blood DNAm likely underlie many of the associations seen in EWAS. Compared to a handful of CpGs previously found to be causally linked with smoking in standard MR studies, we found over 500 CpGs with consistent, nominally significant effects of current smoking on DNAm. These CpGs show broad enrichment for tissue types and functional pathways that implicate numerous well-established harmful health outcomes of smoking, including cell- and neuro-development, carcinogenesis, and immune regulation. In the analyses of former smoking, most of the estimated effects of smoking on DNAm were no longer significant, consistent with the reversibility of smoking’s effects at these loci. Additionally, several CpG sites showed evidence of reverse and possibly bidirectional effects of DNAm on the liability for current smoking, with a subset of these loci enriched for gene regulatory functional elements in the brain. The detection of reverse or bidirectional causal effects of blood DNAm on smoking highlights the potential utility of blood DNAm as a putative biomarker to monitor addiction or interventions.

Previous analyses of smoking-discordant twin pairs in NTR, a subset of the current study sample, found 13 CpG sites with significant DNAm differences between MZ twins discordant for current smoking^39^, suggesting potential causality. In our MR-DoC analyses, eight of the 13 CpGs showed robust evidence of causal effects of current smoking on DNAm, while none showed reverse effects. Taken together, the findings from the two studies further triangulate the evidence for smoking’s effects on DNAm at these sites. Prior summary-statistics-based MR studies have examined causality between *lifetime* (current or former) cigarette smoking and blood DNAm. The MR analyses in GoDMC^18^ did not find evidence of causal effects of lifetime smoking on DNAm, nor *vice versa*. Another study^10^ applied a single MR method and found nominally significant effects of lifetime smoking on DNAm at 11 CpG sites from the Illumina MethylationEPIC array^40^, of which two (cg14580211, cg15212295) overlap with Illumina 450k array data used in the current study. In our MR-DoC analyses, only cg14580211 showed replication in the form of consistent negative causal estimates of current smoking on DNAm. The novel and more extensive causal effects found in our analyses may partly be attributable to the study design’s ability to estimate the causal influences of *current* smoking specifically, as most smoking-associated DNAm changes exhibit substantial reversibility upon smoking cessation^2,21^. Furthermore, the nine CpGs with previously reported reverse effects of DNAm on lifetime smoking behavior (a composite index of initiation, heaviness, and cessation)^11^ in a single MR model showed inconsistent estimates in the three MR-DoC models. Interestingly, two of these CpGs (cg09099830 and cg24033122; both located in gene *ITGAL*) instead showed consistent, nomically significant effects of current smoking on DNAm, underscoring the need for further replication of both prior and current findings.

Of the three CpG sites with robust evidence of DNAm’s effects on current smoking liability, two are located in genes *GNG7* and *RGS3* that are integral to G protein-coupled receptor (GPCR) signaling, adding to the growing literature on GPCR signaling pathways’ potential role in behavioral and neuropsychiatric outcomes^41^. Specifically, differential expressions of both *GNG7*^42^ and *RGS3*^43^ have been associated with addiction-related phenotypes in model organisms. The third CpG annotates to *SLC15A4*, which encodes a lysosomal peptide/histidine transporter involved in antigen presentation and innate immune response^44^, including in mast cells^45^. Thus, DNAm variation at this locus may plausibly reflect individual differences in immunological tolerance of cigarette smoke and, consequently, maintenance of smoking behavior. Interestingly, these CpGs were significantly associated with neither cannabis use^7^ nor alcohol consumption^6^ in recent large-scale EWASs. Notably, though, both these studies reported DNAm associations conditional on cigarette smoking, making them unsuitable for gauging whether the CpGs with putative effects on smoking liability are also associated with other substances. This raises the question of whether cigarette smoking should always be used as a covariate in EWAS. If so, it may be prudent to report supplementary EWAS results without smoking as a covariate, as some CpGs may have a reverse or bidirectional causal relationship with smoking. Note that the EWAS of cannabis use^7^ did perform such preliminary analyses but only reported the results conditional on cigarette smoking.

Several factors need to be considered when interpreting the above results. Although we found relatively few sites with putative effects of whole blood DNAm on smoking liability or with suggestive bidirectional effects, the situation might differ in specific blood cell types or other tissues relevant to smoking, like the brain. The results may also vary in other peripheral tissues, like buccal cells^46^. Moreover, the highly variable predictive strength of mQTL allelic scores across CpG sites (incremental-R^2^ range: 0.43–76.95%; median 4.61%) likely also influenced the power to detect true causal effects of blood DNAm on smoking liability^24^. When considering similar model applications across different health traits, this impact on power is relevant to both directions of causation, as the IV of other traits may not be as strong as the smoking PRS. Additionally, the current study analyzed CpGs from the Illumina 450k array, which covers a small fraction of genome-wide potential methylation sites. Further, many of the measured smoking-associated CpGs lacked a “relevant” mQTL allelic score with F-statistic >10 (Supplementary Figure S35) and so are yet to be tested for *DNAm → Smoking* causal effects. Newer low-cost sequencing technology^47^ may help uncover more such causal relationships in the future.

Like all MR studies, the current results depend on the validity of the IV assumptions^19^, which cannot always be empirically tested. Here, we relied on the statistical significance and consistency of the causal estimates across different specifications of MR-DoC models to account for potential assumption violations, particularly horizontal pleiotropy. Yet, residual bias due to violations of the assumptions underlying MR^19^ and biometrical twin modeling^48^ cannot be ruled out with certainty. Moreover, current MR-DoC models estimated linear causal effects. However, since DNAm is constrained within certain biologically plausible values, the impact of smoking on DNAm may depend on *prior* DNAm. To examine such non-linear causal relationships, MR-DoC with interaction or quadratic effects would be a valuable area of further model development, with numerous potential applications. Finally, we examined causality using only binary smoking-status variables, as the sub-samples restricted to current or former smoking were too small to fit MR-DoC models to smoking quantity (e.g., cigarettes per day) or time since quitting. Further research with larger samples is needed to examine such dose-response causal relationships.

The current study included participants of European ancestry only. Although prior EWASs show highly concordant associations across ancestries^2,7^, examining the generalizability of causal estimates in non-European populations is a critical subject of further research. As MR-DoC models provide causal inference specific to the target dataset, rather than the discovery GWAS samples, future research may apply this study design to subpopulations of interest, e.g., stratified by sex or age (such as children or elderly populations), provided the results from population-wide GWAS generalize adequately. Future applications of MR-DoC analyses to DNAm data may also extend the current work to other health traits and disorders that show robust associations with DNAm and have strong genetic IVs. Recent developments in cost-effective population-scale DNAm microarray technology^49^ can help increase the sample sizes of twin cohorts with DNAm data, enabling wider application of similar causal analyses.

In conclusion, the inability to establish causality is one of the key limitations of EWAS based on surrogate tissues such as blood. Here, we demonstrate an application of the MR-DoC design to examine causality between cigarette smoking and blood DNAm. The results suggest that many of the EWAS associations are likely driven by the causal effects of current smoking on DNAm, though we also find evidence of reverse and potentially bidirectional causal relationships at some sites. Our study highlights the value of integrating DNAm, phenotypic information, and genetic data in twin studies to uncover causal relationships of peripheral blood DNAm with human traits. This study design might be valuable for detecting causal epigenetic biomarkers of (mental) health in general.

## Methods

### Study Sample

The Netherlands Twin Register (NTR) is a community-based twin registry with longitudinal data on health, behaviors, and lifestyle factors, combined with biological samples, including DNA from blood and buccal samples. In the current analyses, we analyzed data from 2,577 individuals participating in the NTR longitudinal surveys^17^ and the NTR biobank project^50^. The study participants comprised 1,730 (67%) female and 847 (33%) male individuals of European genetic ancestry, including 706 monozygotic (MZ) twin pairs, 161 MZ individuals without their co-twin, 412 dizygotic (DZ) twin pairs, and 180 DZ individuals without their co-twin. The participants had both genotypic and epigenome-wide DNAm data and were aged between 18 and 79 years (mean 35.2; S.D. 11.7 years) at the time of blood sample collection.

Previous studies have described the NTR cohort in greater detail^39,51^. NTR genotypic sample and variant quality control (QC), imputation, genetic principal component analysis (PCA), and ancestry-outlier pruning have been described previously^52^. Details specific to the participants included in the present study are included in the Supplementary Methods. Since GoDMC^18^ summary statistics are available for European ancestry only, the current study sample excluded 109 participants identified as European-ancestry outliers in PCA to avoid bias due to ancestry mismatch. The NTR is approved by the Central Ethics Committee on Research Involving Human Subjects of the VU University Medical Centre, Amsterdam, an Institutional Review Board certified by the U.S. Office of Human Research Protections (IRB number IRB00002991 under Federal-wide Assurance- FWA00017598; IRB/institute codes, NTR 98–222, 2003–180, 2008–244). All participants provided written informed consent before data collection.

### Peripheral Blood DNA Methylation and Cell Counts

Epigenome-wide DNAm in peripheral whole blood was measured with the Infinium HumanMethylation450 BeadChip Kit (i.e., the Illumina 450k microarray), following the manufacturer’s protocol^21^. QC and normalization of the DNAm data were performed using a custom pipeline developed by the BIOS (Biobank-based Integrative Omics Study) Consortium, as previously described^51^. Briefly, sample QC was done using MethylAid^53^, followed by probe QC with DNAmArray^54^. The latter removed the probes with a raw signal intensity of zero, bead number <3, or a detection p-value >0.01, as well as the ambiguously mapped probes. Next, samples and probes with >5% missingness were removed. The resulting DNAm data were normalized using the Functional normalization^55^ algorithm implemented in DNAmArray^54^, with the first four PCs (with eigenvalue >1) derived from control probes. Finally, the probes containing a SNP within the CpG site (at C or G nucleotide) were removed regardless of the minor allele frequency. These SNPs were previously identified using DNA sequencing data from the Dutch population^56^. For the current analyses, only autosomal probes were included, yielding 411,169 CpG sites that passed all QC metrics, of which 16,940 sites were reported as associated with current smoking (FDR <0.05) in an independent EWAS^2^. Differential white blood cell counts were also measured in the blood samples to estimate the proportions of neutrophils, lymphocytes, monocytes, eosinophils, and basophils^51^.

Using linear regression models, the normalized β-values of DNAm at each CpG were corrected for commonly used EWAS covariates^57^, including age at blood draw, sex (genotypically inferred biological sex, matched with self-reported gender), measured white blood cell percentages (neutrophils, monocytes, and eosinophils) at blood draw, MH450k array row, and bisulfite sample plate (dummy variables). The residuals from these regression models were standardized (mean = 0, S.D. = 1) and used in MR-DoC models. As in the previous work in this dataset^39^, we did not include lymphocyte percentage as a regression covariate to prevent multicollinearity with neutrophil percentage, while basophil percentage was not included because it had little variation between individuals.

### Cigarette Smoking

Self-reported cigarette smoking status was recorded through an interview during the home visit for blood sample collection in 2004–2008 and 2010–2011. Participants were asked, “Do you smoke?” with one of three possible answers: “No, I never smoked” (N = 1,492), “No, but I did in the past” (N = 549), and “Yes” (N = 528). See Supplementary Methods for the original wording in Dutch. Those endorsing current smoking were asked how many years they had been smoking and how many cigarettes or rolling tobacco they smoked per day. Those endorsing former smoking were asked how many years ago they quit smoking, how many years they had smoked before quitting, and the maximum number of cigarettes or rolling tobacco they used to smoke per day. The responses were checked for consistency with the information from the NTR longitudinal surveys filled out closest to blood sampling. As previously described^58^, potential misclassification of smoking status through self-reports was evaluated based on plasma cotinine levels (a metabolite of nicotine and a biomarker of smoking exposure), measured in a subset of the sample. Of the 591 individuals with self-reported never smoking and measured plasma cotinine, only five (0.8%) had cotinine levels indicative of smoking (≥15 ng/ml), thus suggesting low misclassification of smoking status. The number of individuals endorsing current or former smoking was too small to evaluate a dose-response relationship of the causal effects in MR-DoC models restricted to currently or formerly smoking individuals. Likewise, the sample with former smoking was too small to examine the effect of “time since quitting smoking” on DNAm.

### Instrumental Variables

#### mQTL allelic scores.

We identified 12,940 smoking-associated CpGs with *cis*-mQTL summary statistics available from GoDMC^18^ (excluding NTR), using GoDMC’s definition of “*cis*” interval (within 1Mb of the CpG). In GoDMC, the contributing cohorts performed genome-wide mQTL analyses, testing the associations of ~480,000 CpG sites with ~12 million SNPs. However, before the meta-analysis, the cohort-level results were filtered to retain the SNP-CpG pairs with p < 1 × 10^–5^ within the cohort. Thus, since the summary statistics were already partly thresholded, we computed the mQTL allelic scores by applying clumping and thresholding in *PLINK1.9*^59^. Linkage disequilibrium (LD)-based clumping was performed using --clump-p11 --clump-kb 250, with two levels of LD r^2^ (0.5 and 0.1) specified for --clump-r2, thus yielding two sets of LD-clumped *cis*-SNPs. Using either set of SNPs, we computed the allelic score with --score at a threshold of 0.05 (applied with --q-score-range). If none of the SNPs had p <0.05, no threshold was applied for score calculation. An additional allelic score was calculated using the top *cis*-mQTL (with the minimum association p-value) for each CpG. Thus, for every CpG, three scores were calculated (two LD-clumped mQTL allelic scores, plus the top-mQTL), though these scores were not necessarily distinct; for example, if a CpG had only one *cis*-SNP, all three criteria yielded the same score. Likewise, for some CpGs, the two LD-clumping cut-offs resulted in the same set of SNPs and, hence, identical mQTL allelic scores.

To assess the strength of an mQTL allelic score, we first estimated its incremental R^2^ by fitting generalized estimating equations (GEE), controlling for the standard EWAS covariates (as above), genotyping platform, and the first ten genetic PCs. For each CpG, the effective GEE sample size (*N*_*Eff*_) was computed using the following formulae:

$$N_{Eff}^{MZ}=\frac{2*N_{MZ}}{1+r_{MZ}}$$

$$N_{Eff}^{DZ}=\frac{2*N_{DZ}}{1+r_{DZ}}$$

$$N_{Eff}=N_{Eff}^{MZ}+N_{Eff}^{DZ}+N_{Ind}$$

where, $N_{Eff}^{MZ}$ and $N_{Eff}^{DZ}$ are the estimated effective sample sizes of MZ and DZ twins, *N*_*MZ*_ and *N*_*DZ*_ are the numbers of complete MZ and DZ twin pairs, while *r*_*MZ*_ and *r*_*DZ*_ are the twin phenotypic (DNAm) correlations in MZ and DZ twin pairs, respectively. *N*_*ind*_ is the number of individuals without the co-twin. The estimated effective sample size was then used to transform the incremental R^2^ value into an F-statistic as:

$$F=\frac{R^{2}}{1-R^{2}}\times \frac{N_{Eff}-K}{K-1}$$

where *K* = 2, given two parameter estimates: the intercept and the regression coefficient of the mQTL allelic score.

#### PRS of Regular Smoking Initiation.

We used European-ancestry GWAS summary statistics for smoking initiation (i.e., initiation of regular smoking) from the GWAS & Sequencing Consortium of Alcohol and Nicotine use (GSCAN; excluding NTR)^20^ to compute the PRS of smoking in NTR using *LDpred v0.9*^60^. Of note, the phenotypic definition in the GWAS (smoking initiation = current/former versus never smoking) was different from the smoking phenotypes (current versus never and former versus never smoking) in the MR-DoC models. However, in these causal models, the strength of the IV, the extent of horizontal pleiotropy with DNAm, and the estimated causal effects on DNAm are specific to the smoking phenotype used in the models. As a result, this approach allowed us to assess the causal relationships of DNAm with current and former smoking separately. See Supplementary Methods for a detailed description of PRS calculation and estimation of incremental R^2^. Using linear regression models, we residualized the PRS of smoking and all mQTL allelic scores for the genotyping platform and the first ten genetic PCs. The residuals were scaled to have a mean of zero and a variance of one before being included as IVs in MR-DoC models.

### MR-DoC Models

Causal inference in the twin *Direction-of-Causation* models uses the differences in cross-twin cross-trait correlations under different directions of causation to identify the model that fits the data best^15^. On the other hand, MR analyses rely on three assumptions of a valid IV^3,19^, that the IV is (1) associated with the exposure (“relevance”), (2) not correlated with any omitted confounding variables (“exchangeability”), and (3) independent of the outcome, given the exposure (“exclusion restriction”). Here, we used the criterion of F-statistic >10 to define the “relevance” of the IV. Further, germline genetic variants are often assumed to satisfy the “exchangeability” assumption due to Mendel’s laws of random segregation and independent assortment. The “exclusion restriction” assumption for a genetic IV implies no horizontal pleiotropy with the outcome. As described above, we relied on different specifications of MR-DoC models to account for potential horizontal pleiotropy. MR-DoC1 model allowed estimating and controlling for horizontal pleiotropy from the IV to the outcome, though it required us to fix the unique environmental confounding at a specific value (here, zero)^13^. MR-DoC2 model leverages the covariance between two polygenic or multiallelic IVs, beyond the bidirectional causal effects, to partly accommodate horizontal pleiotropy^14^.

We used the *OpenMx* (version 2.21.8)^61^ package in R (version 4.3.2) to fit the MR-DoC models, using the code provided in the original publications^13,14^. Binary smoking status was examined under the liability threshold model^62^, assuming a latent liability distribution with its mean fixed at zero and variance fixed at one, while the threshold was freely estimated.

Before fitting the MR-DoC models, we examined univariate ACE twin models of smoking status to estimate the additive genetic (A), shared environmental (C), and unique environmental (E) variance components of the latent liability scale, with age and sex as covariates. Maximum-likelihood tetrachoric correlation estimates for current versus never smoking were: *r*_*MZ*_ = 0.925 (*S*. *E*. = 0.021) in MZ pairs and *r*_*DZ*_ = 0.533 (*S*. *E*. = 0.083) in DZ pairs. Likewise, former versus never smoking had *r*_*MZ*_ = 0.822 (*S*. *E*. = 0.038) and *r*_*DZ*_ = 0.474 (*S*. *E*. = 0.096). Based on likelihood-ratio tests (LRT), an AE twin model was the most parsimonious model for both current versus never (AE versus ACE LRT *p* = 0.417) and former versus never smoking (AE versus ACE LRT *p* = 0.530) (Supplementary Table S31). The estimated variance components of current versus never smoking liability were *A* = 0.927 (maximum-likelihood 95% confidence interval: 0.879, 0.959) and *E* = 0.073 (0.041, 0.121). The corresponding estimates of former versus never smoking were *A* = 0.827 (0.745, 0.888) and *E* = 0.173 (0.112, 0.255).

Prior twin analyses of DNAm at CpG sites in NTR^51^ showed that, of the 411,169 autosomal post-QC CpG sites, the AE twin model was the best fitting model at all but 426 sites, with significant (after multiple-testing correction of LRT p-values) C variance at 185 sites and significant non-additive genetic (D) variance at 241 sites. Of the smoking-associated CpGs^2^, only two CpGs had significant estimates of C, while only seven CpGs had significant estimates of D. Thus, in MR-DoC models, we specified an AE variance decomposition of DNAm at all smoking-associated CpGs. Note that, in the results presented above, none of the CpG sites with consistent, nominally significant estimates of causal effects in either direction (525 sites with *current smoking → DNAm*; 64 sites with *DNAm → current smoking*) have significant C or D estimates per the previous univariate twin analyses^51^. Moreover, since smoking status liability also has an AE variance decomposition, including a C or D variance component of DNAm in the model would not change the possible sources of covariance between smoking status and DNAm in the model.

We fitted five sets of MR-DoC models with current versus never smoking and similar sets with former versus never smoking (Figure 1): (1) *Smoking → DNAm* MR-DoC1 with horizontal pleiotropy, (2) *Smoking → DNAm* MR-DoC1 with unique environmental confounding, (3) *DNAm → Smoking* MR-DoC1 with horizontal pleiotropy, (4) *DNAm → Smoking* MR-DoC1 with unique environmental confounding, and (5) bidirectional MR-DoC2. Each model included age and sex as covariates of smoking status. In each model, the residual variance of smoking status liability is decomposed into $a_{S}^{2}$ (A) and $e_{S}^{2}$ (E), while that of DNAm is decomposed into $a_{D}^{2}$ (A) and $e_{D}^{2}$ (E). The correlation between the latent A factors of smoking and DNAm (rA) represents the confounding due to additive genetic factors. The correlation between the latent E factors (rE) represents the confounding due to unique environmental factors. Across all models, the causal path from smoking to DNAm is labeled g_1_, while that from DNAm to smoking is labeled g_2_. The residualized PRS and mQTL allelic scores are regressed on respective latent factors, representing the underlying “true” standardized scores with mean fixed at zero and variance fixed at one. The coefficient of the path from the latent score to the observed score estimates the standard deviation of the observed score (*SD*_*PRS*_ and *SD*_*mQTL*_, respectively).

Thus, for each CpG site included in the analyses, three causal estimates were obtained in either direction (*Smoking → DNAm*, or *DNAm → Smoking*) from (1) MR-DoC1 with horizontal pleiotropy, (2) MR-DoC1 with unique environmental confounding, and (3) MR-DoC2. For each set of causal estimates across CpG sites, we calculated the Bayesian inflation factor (λ) using the R package *bacon*^23^, made QQ plots using the R package *GWASTools*^63^, and then applied Benjamini-Hochberg FDR correction^64^ to the p-values using the R package *qvalue*^65^. For Bonferroni multiple-testing correction, the significance level was defined as *α* = 0.05/16940 = 2.95 × 10^−6^ for *Current Smoking → DNAm* MR-DoC1 models and *α* = 0.05/11124 = 4.49 × 10^−6^ for *DNAm → Current Smoking* MR-DoC1 and bidirectional current-smoking MR-DoC2 models.

### Functional Enrichment Analyses

We used Metascape^25^ (v3.5.20240101; https://metascape.org/gp/index.html#/main/step1, with the default settings for “Express” analyses) to perform gene-set annotation and functional enrichment analyses of the CpGs with potential causal effects in either direction. The input list of gene IDs was selected based on proximity to the CpGs with consistent and nominally significant (p <0.05) estimates in all three models; i.e., 64 CpGs with potential *DNAm → Current Smoking* effects (“Nearest Gene” in Supplementary Table S3) and 525 CpGs with potential *Current Smoking → DNAm* effects (“Nearest Gene” in Supplementary Table S1). None of the sites with potential *DNAm → Current Smoking* effects are located in the MHC region. For *Current Smoking → DNAm* effects, 21 additional sites in the MHC region showed consistent, nominally significant estimates. There was no significant relationship between a CpG site having consistent causal estimates and its being located in the MHC region (Fisher’s exact test p-value = 0.5455). However, out of an abundance of caution, the sites located in this region were not included in the enrichment analyses to avoid sites with potentially unreliable results due to its complex LD structure.

As described in the Metascape manuscript^25^, the program performed integrated enrichment analyses against multiple reference ontology knowledgebases, including GO processes^66^, KEGG pathways^67^, canonical pathways^68^, and Reactome gene sets^69^. The significant terms with a hypergeometric p-value <0.01 and >1.5-fold enrichment were clustered into a hierarchical tree based on Kappa-statistical similarities among their gene memberships. The tree was then cast into clusters based on a threshold of 0.3 kappa score to obtain enriched, non-redundant ontology terms.

### eFORGE (experimentally derived Functional element Overlap analysis of ReGions from EWAS)

We performed *eFORGE 2.0*^26,27,70^ analyses of the selected CpG probe IDs with consistent and nominally significant (p <0.05) estimates in either direction (from Supplementary Tables S1, S3). Using the web-based tool (https://eforge.altiusinstitute.org/), we examined the overlap between the implicated CpGs and multiple comprehensive reference sets of genomic and epigenomic features that regulate gene expression in different tissues and cell types. The platform was set as “Illumina 450k array”, with default analysis options: proximity = 1kb window, background repetitions = 1000, and significance thresholds of FDR <0.01 (strict) and FDR <0.05 (marginal). Three sets of analyses were performed for each list of probe IDs, selecting the reference data from “Consolidated Roadmap Epigenomics - Chromatin - All 15-state marks”, “Consolidated Roadmap Epigenomics - DHS”, and “Consolidated Roadmap Epigenomics - All H3 marks”.

The eFORGE results include the specific probe IDs overlapping between the input set and the reference sample. We performed iterative follow-up analyses for the CpGs with potential *DNAm → Current Smoking* effects, based on the overlapping probe IDs to examine the specificity of significant (FDR <0.01) enrichment in tissues of interest. Analyses restricted to the 21 CpGs overlapping with enhancers in the fetal brain (Supplementary Figure S18, Table S12) showed significant enrichment only for enhancers in the fetal brain samples, suggesting high specificity (Supplementary Figure S21). The histone mark analyses also showed enrichment in the fetal brain (though not specific to the brain), wherein all 21 CpGs overlapped with H3K4me1, while a subset of 17 CpGs overlapped with H3K4me3 (Supplementary Figure S22). Finally, we performed analyses restricted to these 17 CpGs.

We performed similar follow-up analyses with probe IDs showing overlap with enhancers in the lung (potentially etiologically relevant tissue) and the primary B-cells in cord blood (the tissue type with the most significant enrichment) (from Supplementary Figure S18, Table S12). We also examined the overlap between the CpGs with potential *DNAm → Current Smoking* effects and the genes implicated in the GWAS of blood cell counts^37^ to probe the potential impact of the cell-count GWAS associations on the causal inference and cell-type enrichment. Similar overlap was examined for the subset of CpGs overlapping with enhancers in cord blood primary B cells.

## Supplementary Material

Supplement 1

Supplement 2

Supplement 3

## Data Availability

Data from the Netherlands Twin Register (NTR) may be accessed for research purposes by submitting a data-sharing request. Further information about NTR data access is available at https://ntr-data-request.psy.vu.nl/. Results of all MR-DoC models fitted in this study are available as Supplementary Data.
