# Supplementary material for: Twin-based Mendelian Randomization Analyses Highlight Smoking’s Effects on Blood DNA Methylation, with Putative Reverse Causation
Source: medRxiv. 2024 Jun 20:2024.06.19.24309184. Preprint. [Version 1] doi: 10.1101/2024.06.19.24309184 (PMC11213072; doi:10.1101/2024.06.19.24309184)
Supplement: Supplementary file 1 [file media-1.pdf]

Supplementary Information

Twin-based Mendelian Randomization Analyses Highlight Smoking's Effects on Blood DNA Methylation, with Putative Reverse Causation

Madhurbain Singh[1,2,3]*, Conor V. Dolan[3,4], Dana M. Lapato[1,2], Jouke-Jan Hottenga[3,4], René Pool[3,4], Brad Verhulst[5], Dorret I. Boomsma[3,4,12], Charles E. Breeze[6,7], Eco J. C. de Geus[3,4], Gibran Hemani[8], Josine L. Min[8], Roseann E. Peterson[9,10,1], Hermine H. M. Maes[1,2], Jenny van Dongen[3,4,11]*, and Michael C. Neale[1,2,3,11]*

1. Virginia Institute for Psychiatric and Behavioral Genetics, Department of Psychiatry, Virginia Commonwealth University, Richmond, VA, USA
2. Department of Human and Molecular Genetics, Virginia Commonwealth University, Richmond, VA, USA
3. Department of Biological Psychology, Vrije Universiteit (VU) Amsterdam, Amsterdam, The Netherlands
4. Amsterdam Public Health Research Institute, Amsterdam, The Netherlands
5. Department of Psychiatry and Behavioral Sciences, Texas A&M University, College Station, TX, USA
6. Division of Cancer Epidemiology and Genetics, National Cancer Institute, National Institutes of Health, Department Health and Human Services, Bethesda, MD, USA
7. UCL Cancer Institute, University College London, London, UK.
8. MRC Integrative Epidemiology Unit, University of Bristol, Bristol, UK
9. Department of Psychiatry and Behavioral Sciences, SUNY Downstate Health Sciences University, Brooklyn, NY, USA
10. Institute for Genomics in Health, SUNY Downstate Health Sciences University, Brooklyn, NY, USA

11. These authors jointly supervised this work.
12. Current address: Department of Complex Trait Genetics, Center for Neurogenomics and Cognitive Research, Vrije Universiteit (VU) Amsterdam, Amsterdam, The Netherlands

*Corresponding authors:
Madhurbain Singh. Email: singhm18@vcu.edu. Address: Virginia Institute for Psychiatric and Behavioral Genetics, 800 E. Leigh St., Suite 100, Richmond, VA 23298, USA
Jenny van Dongen. Email: j.van.dongen@vu.nl. Address: Department of Biological Psychology, Vrije Universiteit Amsterdam, van der Boechorststraat 7, 1081 BT Amsterdam, The Netherlands
Michael C. Neale. Email: michael.neale@vcuhealth.org. Address: Virginia Institute for Psychiatric and Behavioral Genetics, 800 E. Leigh St., Suite 100, Richmond, VA 23298, USA

Table of Contents

Supplementary Methods *4*

Genotypic Data, Principal Components Analysis, and Ancestry Outlier Pruning **4**

Smoking Assessment at Blood Sampling **4**

Polygenic Risk Score of Smoking **4**

Supplementary Figures *6*

Figure S1 .... **6**

Figure S2 .... **6**

Figure S3 .... **7**

Figure S4 .... **7**

Figure S5 .... **8**

Figure S6 .... **9**

Figure S7 .... **10**

Figure S8 .... **11**

Figure S9 .... **12**

Figure S10 .... **13**

Figure S11 .... **14**

Figure S12 .... **16**

Figure S13 .... **17**

Figure S14 .... **18**

Figure S15 .... **19**

Figure S16 .... **20**

Figure S17 .... **20**

Figure S18 .... **21**

Figure S19 .... **22**

Figure S20 ....**23**

Figure S21 ....**24**

Figure S22 ....**25**

Figure S23 ....**26**

Figure S24 ....**27**

Figure S25 ....**28**

Figure S26 ....**29**

Figure S27 ....**30**

Figure S28 ....**31**

Figure S29 ....**32**

Figure S30 ....**33**

Figure S31 ....**34**

Figure S32 ....**35**

Figure S33 ....**36**

Figure S34 ....**37**

Figure S35 ....**38**

References...***39***

Supplementary Methods

In this study, we analyzed data from the Netherlands Twin Register (NTR)[1] to examine the causal influences between smoking status and blood DNA methylation (DNAm) using MR-DoC models[2,3]. In the current analyses, we included data from European-ancestry adult twins with both genotypic and DNAm data, comprising 2,577 individuals (67% female).

Genotypic Data, Principal Components Analysis, and Ancestry Outlier Pruning

The DNA samples included in the current study were genotyped on 3 SNP (single nucleotide polymorphism) microarray platforms: Affymetrix 6.0 (N= 2,399), Affymetrix Axiom (N= 83), and Illumina GSA NTR array (N= 95). Genotype calling was done following the manufacturer's protocols. Sample and variant quality control (QC), imputation, genetic principal component analysis (PCA), and ancestry assignment have been previously described[4]. Briefly, after QC and harmonizing variants across the three platforms, the data were aligned to the positive strand of *Genome Reference Consortium Human Build 37* (GRCh37) and then imputed against the European (EUR) super-population of the 1000 Genomes Project Phase-3 (KGP3)[5], the Haplotype Reference Consortium (HRC)[6] 1.1 (Ega version), and the Genome of the Netherlands Consortium (GoNL)[7] reference panels. Using SmartPCA in EIGENSTRAT[8], the first 20 PCs for the genotypic data were calculated in the KGP3 data, and the NTR samples were then projected onto the PC space based on the SNP weights. Samples identified as outliers in the PC space were then excluded.

Smoking Assessment at Blood Sampling

At the time of blood sampling for DNAm assessment, the participants answered the following questions about smoking in Dutch. The English translations are described in the main **Methods** section.

Rookt u?	1. Ja	1a: hoelang rookt u al? ……………….…..jaar
		1b: hoeveel sigaretten/ shagjes per dag? ……………………………..sigaretten/ shagjes
	2. Nee, wel in het verleden	2a: hoelang is dat geleden?.........................jaar
		2b: hoeveel jaren heeft u gerookt?...............jaar
		2c: hoeveel rookte u per dag (max)? ...…………………….……….sigaretten/ shagjes
	3. Nooit	
Gerookt binnen laatste uur voor bloedafname?	1. Ja<br>2. Nee<br>3. Nvt	

Polygenic Risk Score of Smoking

The PRS of smoking was based on the European-ancestry summary statistics from the genome-wide association study (GWAS) of smoking initiation (lifetime regular smoking) by GSCAN (GWAS & Sequencing Consortium of Alcohol and Nicotine use)[9], excluding the NTR from the meta-analysis.

As described in a previous study using the same PRS in the NTR[4], the post-imputation SNPs from the merged best-guess three-platform data were QCed to satisfy the following criteria: MAF >0.01,

HWE p >0.00001, Mendel error rate < 1%, and genotype call rate over 98%. Furthermore, the imputation info for the three platforms needed to be above 0.10, and the allele frequency between platforms after imputation could not differ more than 2%, leaving a total of 7,551,860 post-QC SNPs for analysis. The PRS was calculated using *LDpred* v0.9[10], with HRC+GoNL as the LD (linkage disequilibrium) reference panel. For estimating the target LD structure, we used a subset of unrelated individuals and a set of well-imputed variants in the NTR. The parameter `ld_radius` was set by dividing the number of variants in common (from the output of the coordination step) by 12000. For the coordination step, the median sample size was used as the input value for N. For the LDpred step, we applied the following thresholds for the fraction of variants with non-zero effects (in addition to the default infinitesimal model): `--PS=0.5,0.3,0.2,0.1,0.05,0.01`.

To determine the LDPred threshold that yielded the PRS with the highest predictive power for the variables of interest (current vs. never and former vs. never smoking), we fitted logistic regression models in R (v4.3.2) to estimate incremental R^2 on a liability scale. We first fitted a null logistic regression model using the `glm()` function with `family=binomial(link='logit')` and a standard set of covariates comprising age (linear and quadratic), sex, SNP microarray platform (dummy variables), and the first ten genetic PCs (without including the PRS). Then, we fitted a full model with the PRS as an additional independent variable. We estimated the liability-scale R^2 in both models and then the difference in the two R^2 estimates as the variance in the outcome variable explained by the PRS (controlling for the covariates). For both outcome variables (current and former smoking), the PRS with the highest incremental R^2 was based on a threshold of 0.1 and thus retained for further analyses. The PRS was residualized for the SNP microarray platform and the first ten genetic PCs using linear regression models. The residuals were then standardized to have a mean of zero and an S.D. of one before using it as an IV in the MR-DoC models.

Supplementary Figures

Figure S1

QQ Plot of MR-DoC1 models (with unique environmental confounding, rE) of Current Smoking → DNAm at 411,169 epigenome-wide CpGs (Bayesian genomic inflation factor, λ = 1.09).

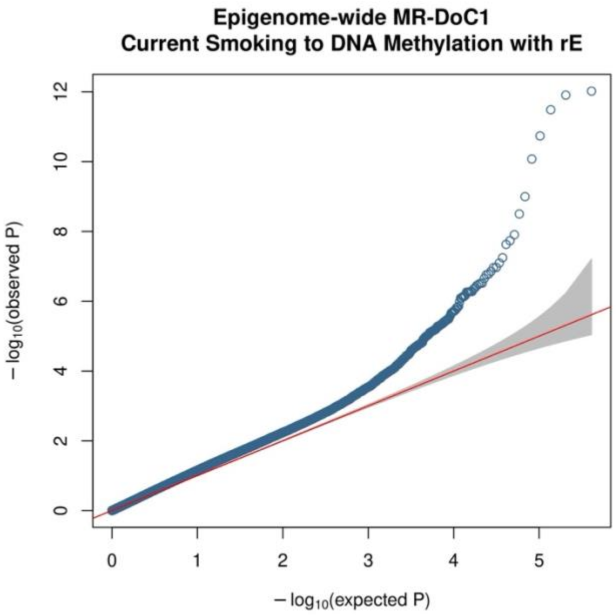

Figure S2

QQ Plot of MR-DoC1 models (with unique environmental confounding, rE) of Current Smoking → DNAm at 16,940 smoking-associated CpGs (Bayesian genomic inflation factor, λ = 1.20).

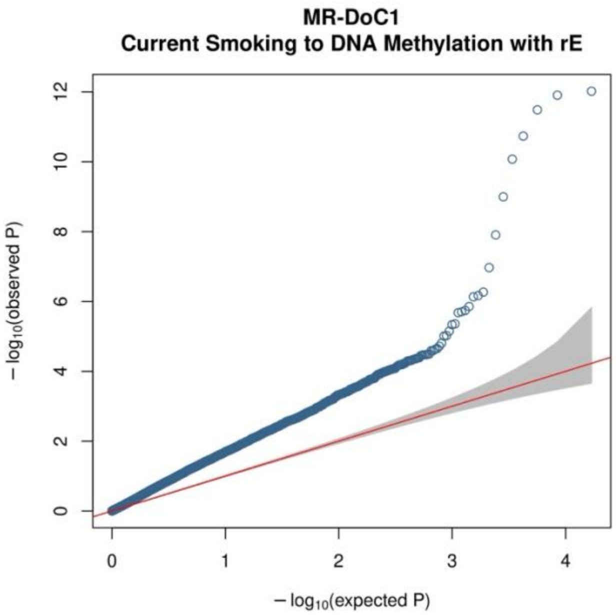

Figure S3

QQ Plot of the Current Smoking → DNAm causal estimates in MR-DoC2 models across 11,124 smoking-associated CpGs (Bayesian genomic inflation factor, λ = 1.20).

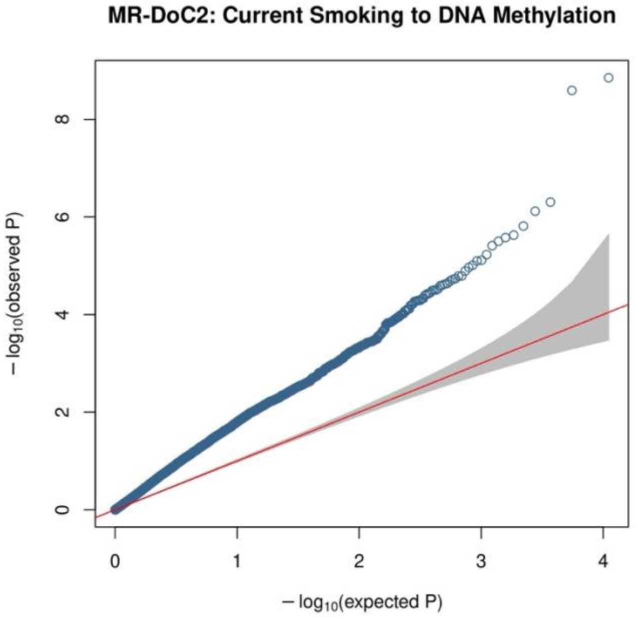

Figure S4

QQ Plot of the DNAm → Current Smoking causal estimates in MR-DoC2 models across 11,124 smoking-associated CpGs (Bayesian genomic inflation factor, λ = 1.01).

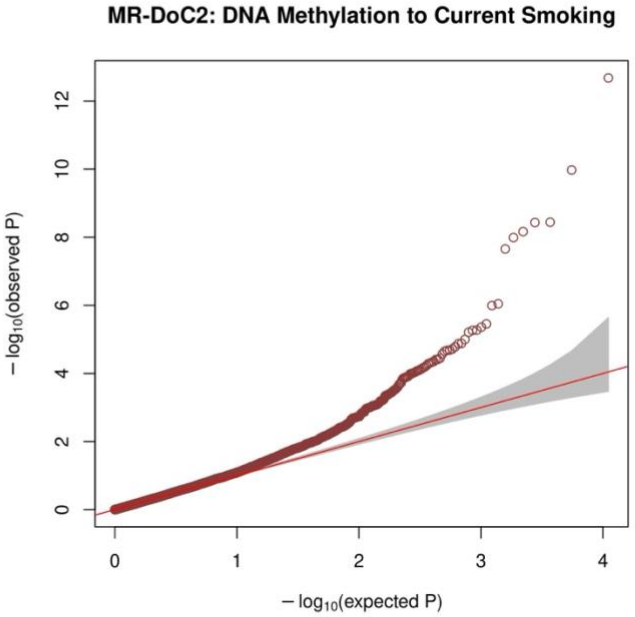

Figure S5

Bidirectional Causal Estimates at the 64 CpGs with Robust Evidence of the Causal Effects of Current Smoking on DNA methylation

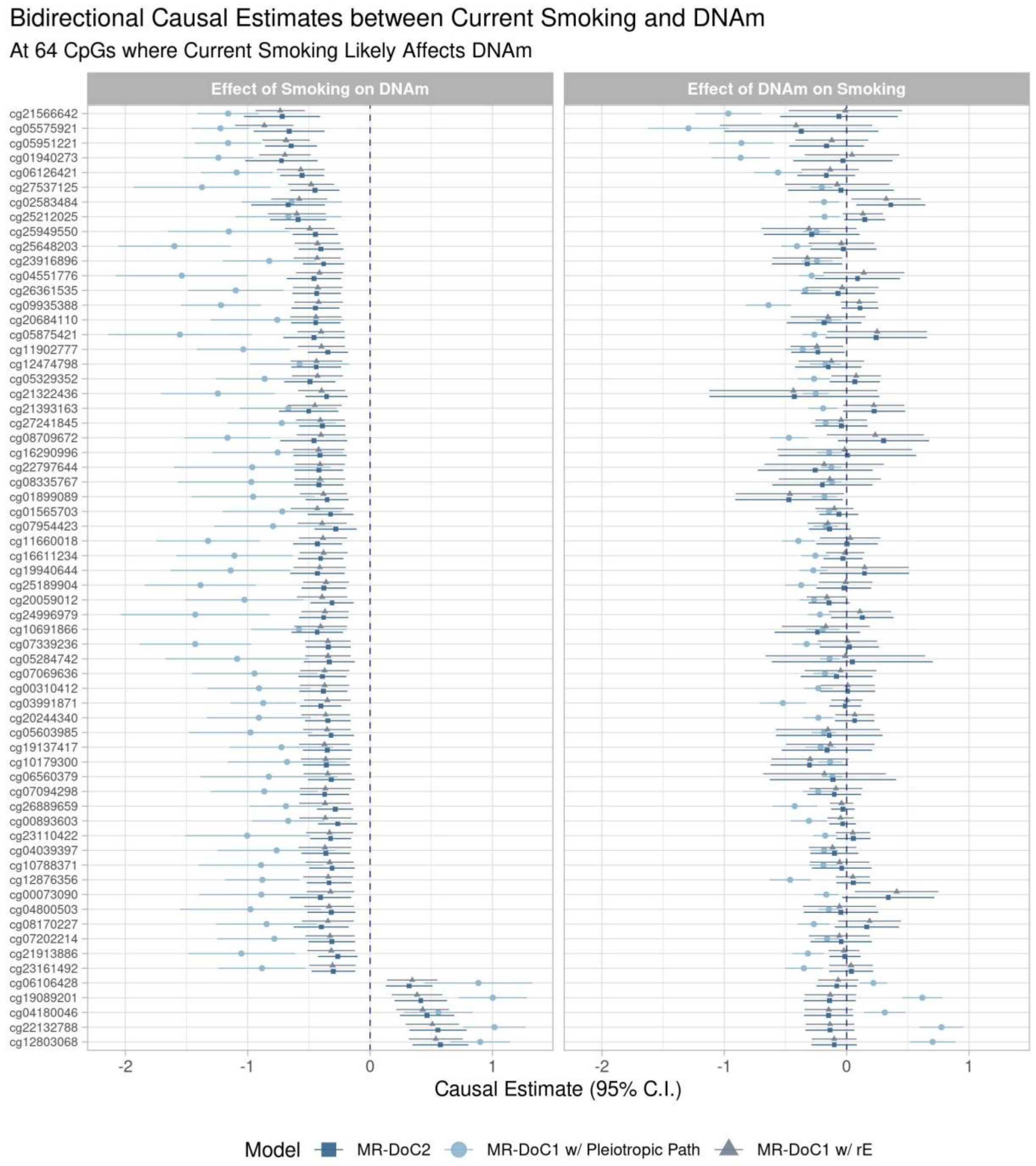

These CpGs did not show robust evidence for the reverse effects of DNAm on current smoking. Please refer to **Supplementary Tables S1** (*Current Smoking* → *DNAm*) and **S2** (*DNAm* → *Current Smoking*) for the corresponding data.

Figure S6

Upset plot of the intersection of CpGs with statistically significant Current Smoking → DNAm effects after Bonferroni correction in each of the three MR-DoC models

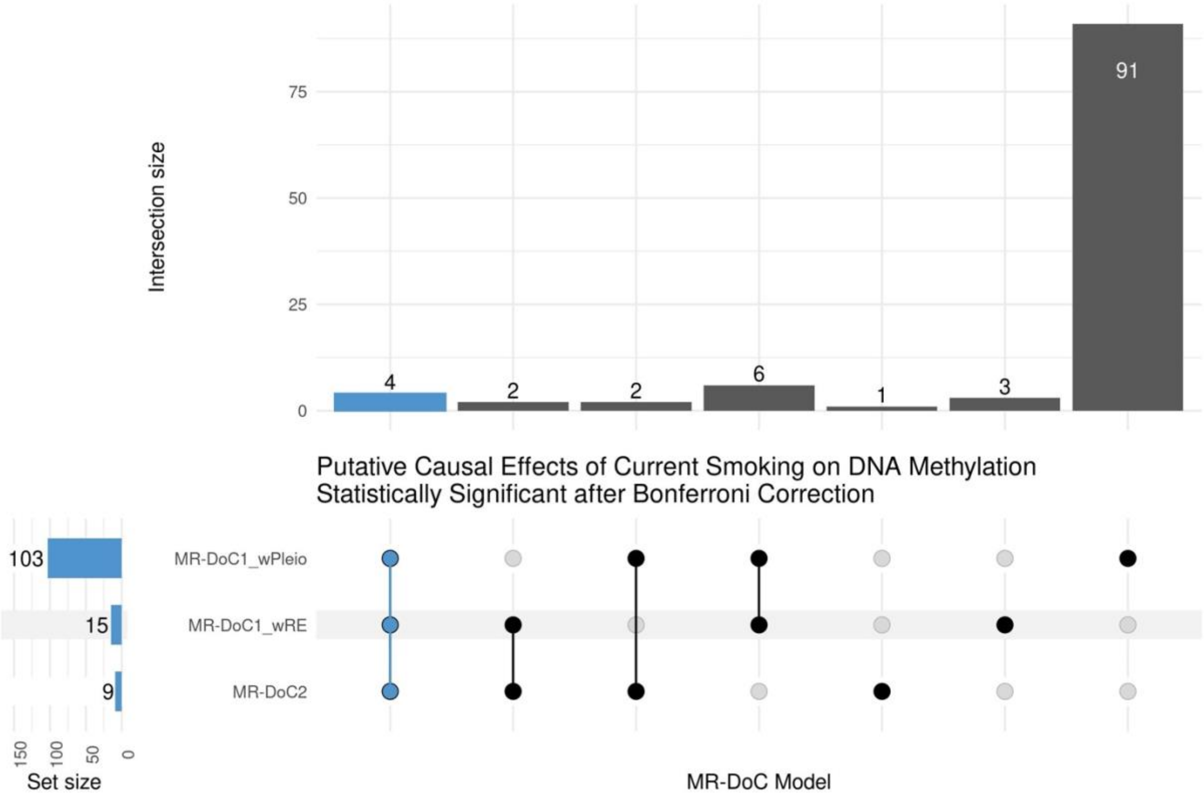

Note. Please refer to **Supplementary Table S1** for the corresponding data

Figure S7

Upset plot of the intersection of CpGs with statistically significant DNAm → Current Smoking effects after Bonferroni correction in each of the three MR-DoC models

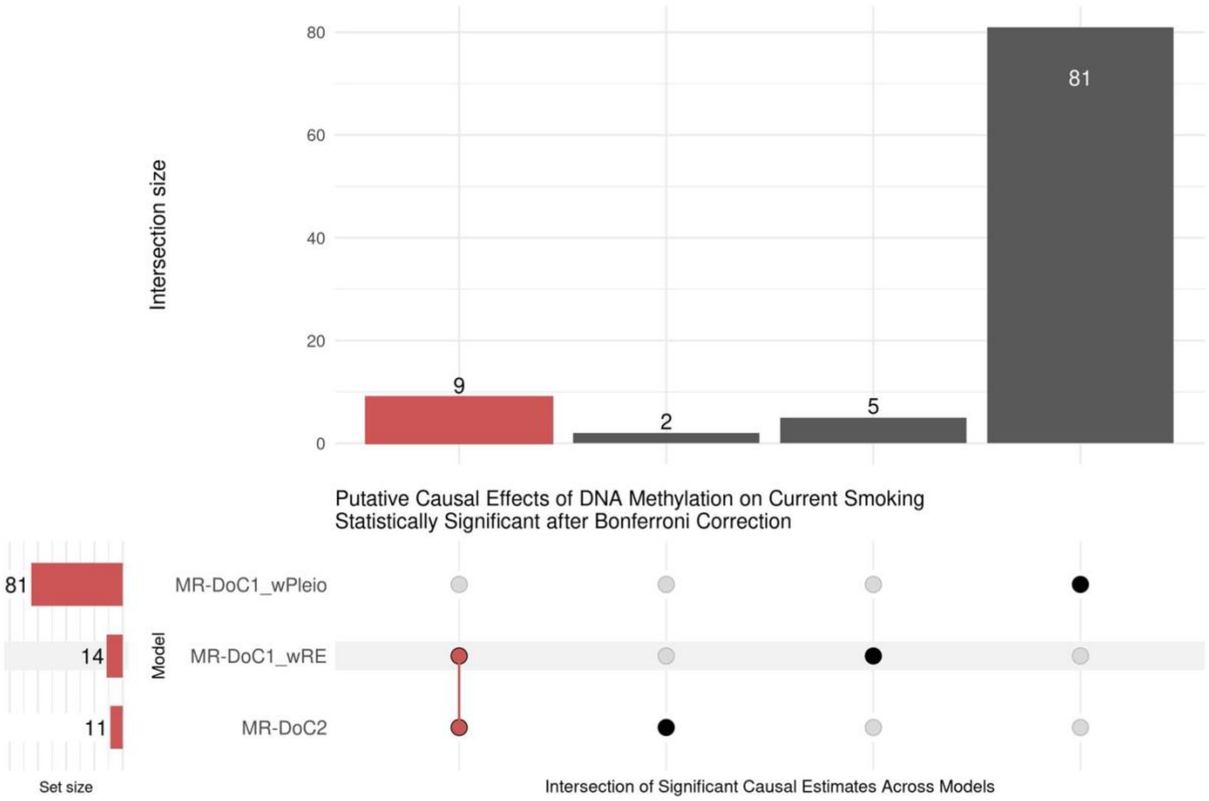

Note. Please refer to **Supplementary Table S3** for the corresponding data

Figure S8

19 CpGs with potential bidirectional causal effects between current smoking and DNA methylation

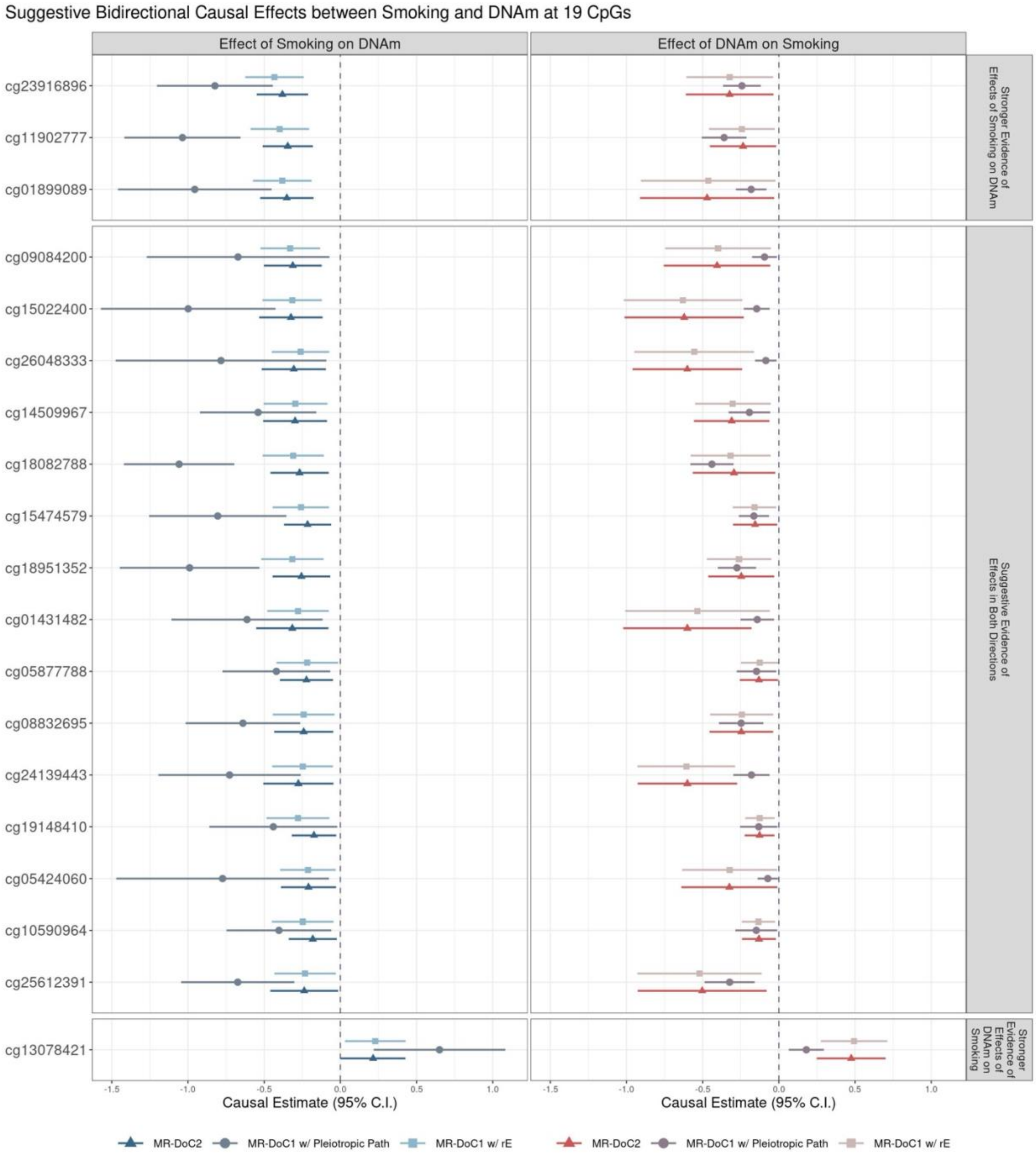

Note. Three CpGs had more robust evidence of *Current Smoking* → *DNAm* causal effects than *vice versa*. One CpG had more robust evidence of *DNAm* → *Current Smoking* causal effects than *vice versa*. The rest 15 CpGs had only suggestive evidence (consistent, nominally significant estimates across models) in both directions. Please refer to **Supplementary Tables S1-S4** for the corresponding data.

Figure S9

Top Enriched Ontology Clusters in Metascape's Gene Annotation and Functional Enrichment Analyses of the 525 CpGs (outside the MHC region) with Potential Current Smoking → DNAm effects

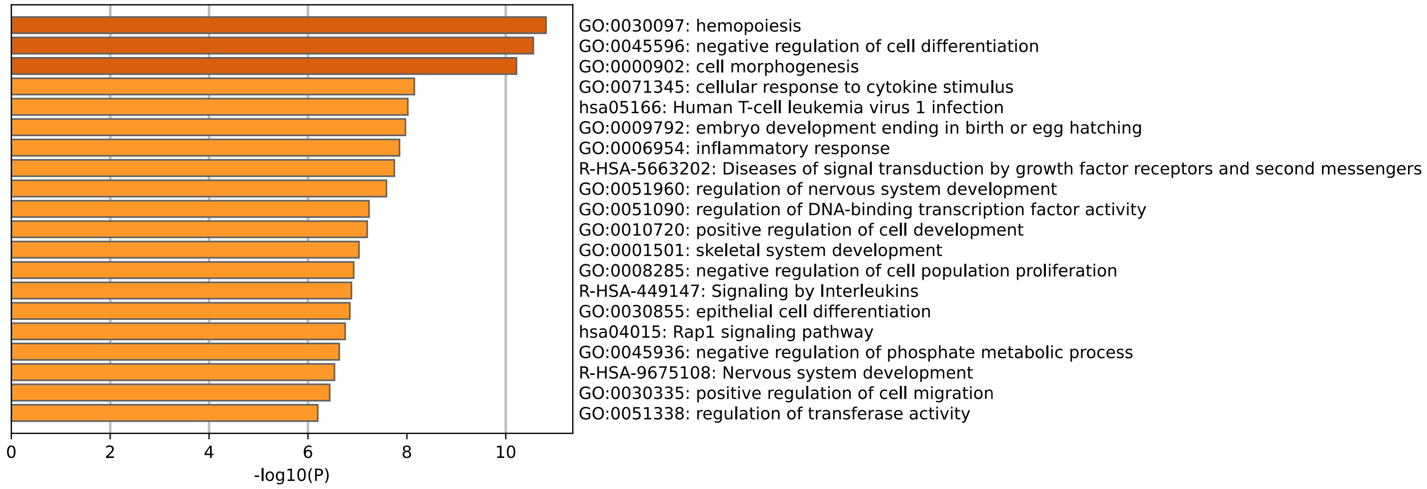

Note. The "NearestGene" IDs from *Supplementary Table S1* were used as the input data for Metascape[12]. Please refer to **Supplementary Tables S5-S6** for the corresponding annotation and enrichment results. As detailed in the Metascape manuscript[12], the program first identified all significant ontology terms, including GO/KEGG terms, canonical pathways, and hallmark gene sets. The significant terms (based on hypergeometric p-value <0.01 and >1.5-fold enrichment) were then clustered into a hierarchical tree based on Kappa-statistical similarities among their gene memberships. The tree was then cast into term clusters based on a threshold of 0.3 kappa score. The enrichment clusters and their underlying terms are marked as "Summary" and "Membership", respectively, under the column *GroupID* in *Supplementary Table S24*. The "Summary" terms provide an overview of enriched, non-redundant ontology terms.

Figure S10

Enrichment Results for Gene-Ontology (GO) Processes in Metascape's Gene Annotation and Functional Enrichment Analyses of the 525 CpGs (outside the MHC region) with Potential Current Smoking → DNAm effects

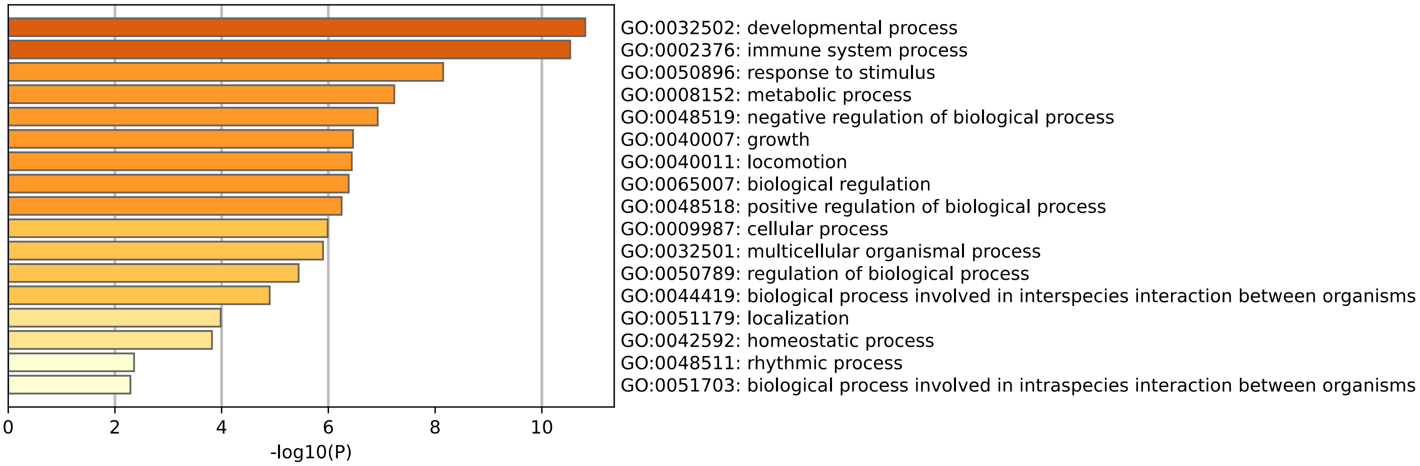

Note. The "NearestGene" IDs from *Supplementary Table S1* were used as the input data for Metascape[12]. Please refer to **Supplementary Table S6** for all enrichment results.

Figure S11

Top 100 Ontology Terms in Metascape's Gene Annotation and Functional Enrichment Analyses of the 525 CpGs (outside the MHC region) with Potential Current Smoking → DNAm effects

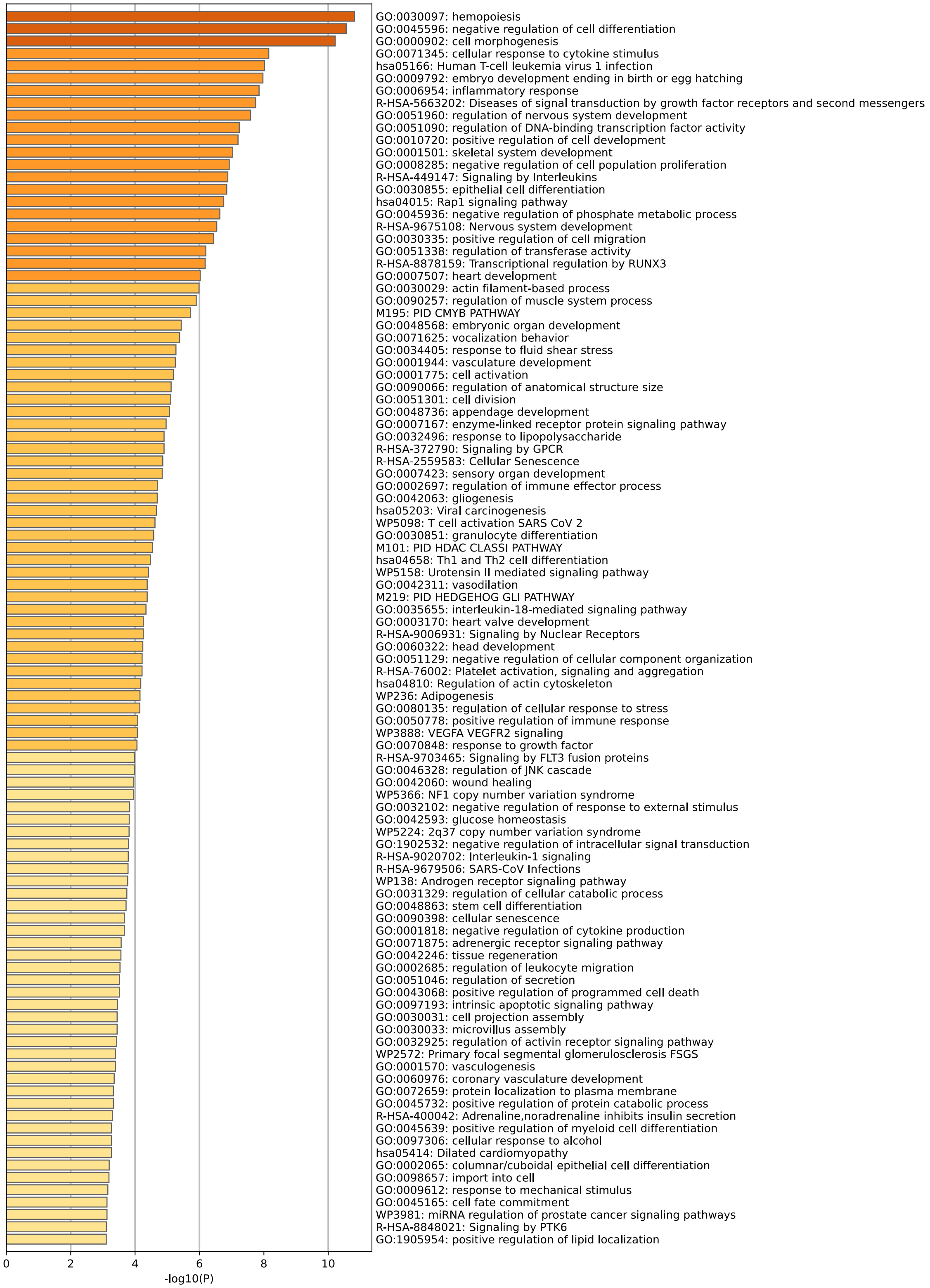

Note. The “NearestGene” IDs from *Supplementary Table S1* were used as the input data for Metascape[12]. Please refer to **Supplementary Table S6** for all enrichment results.

Figure S12

eFORGE analyses of overlap between gene-regulatory chromatin states and the 525 CpGs (outside the MHC region) with potential Current Smoking → DNAm effects

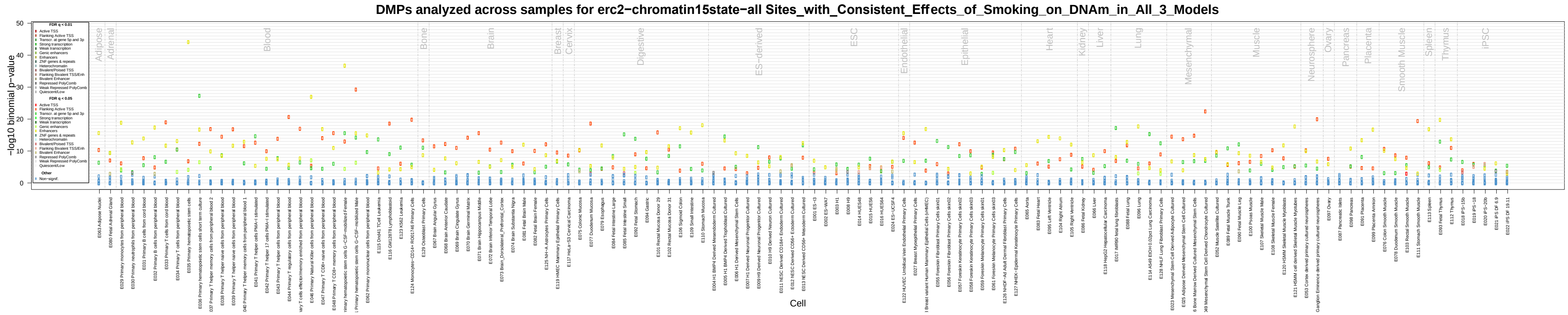

Note. Please refer to **Supplementary Table S7** for the corresponding data.

Figure S13

eFORGE analyses of overlap between histone-mark modifications *and the 525 CpGs (outside the MHC region) with potential Current Smoking* → *DNAm effects*

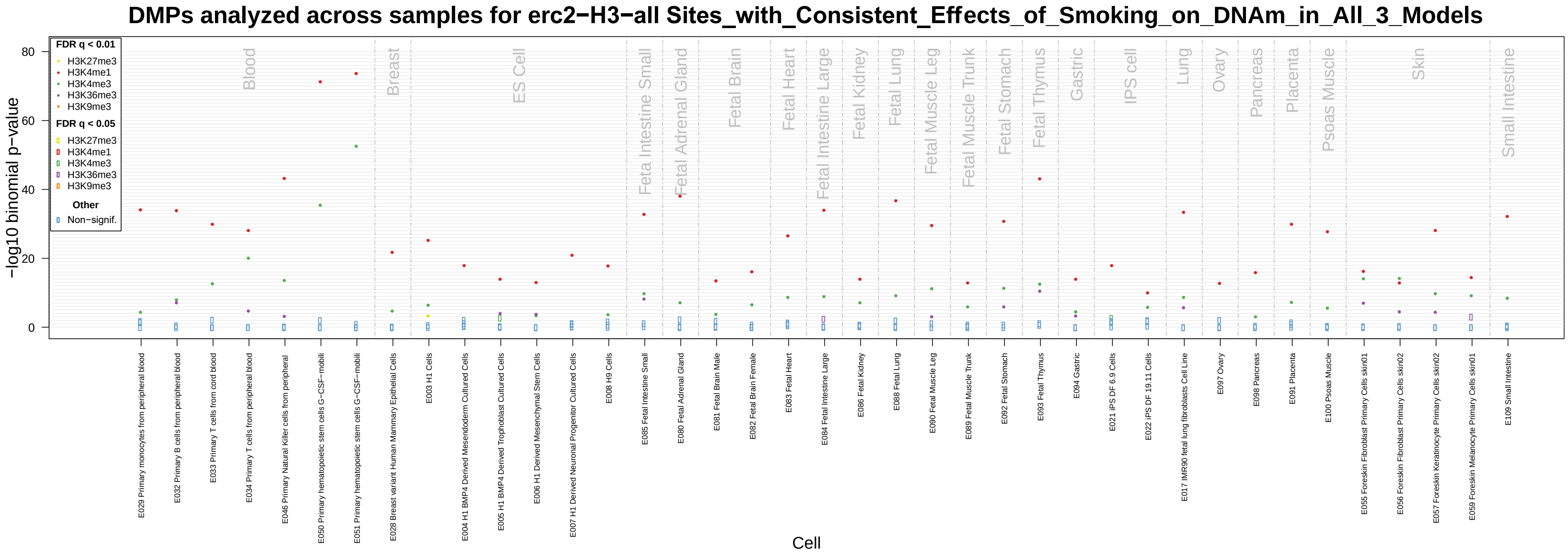

Note. Please refer to **Supplementary Table S8** for the corresponding data.

Figure S14

eFORGE analyses of overlap between DNase hypersensitivity (DHS) sites and the 546 CpGs with potential Current Smoking → DNAm effects

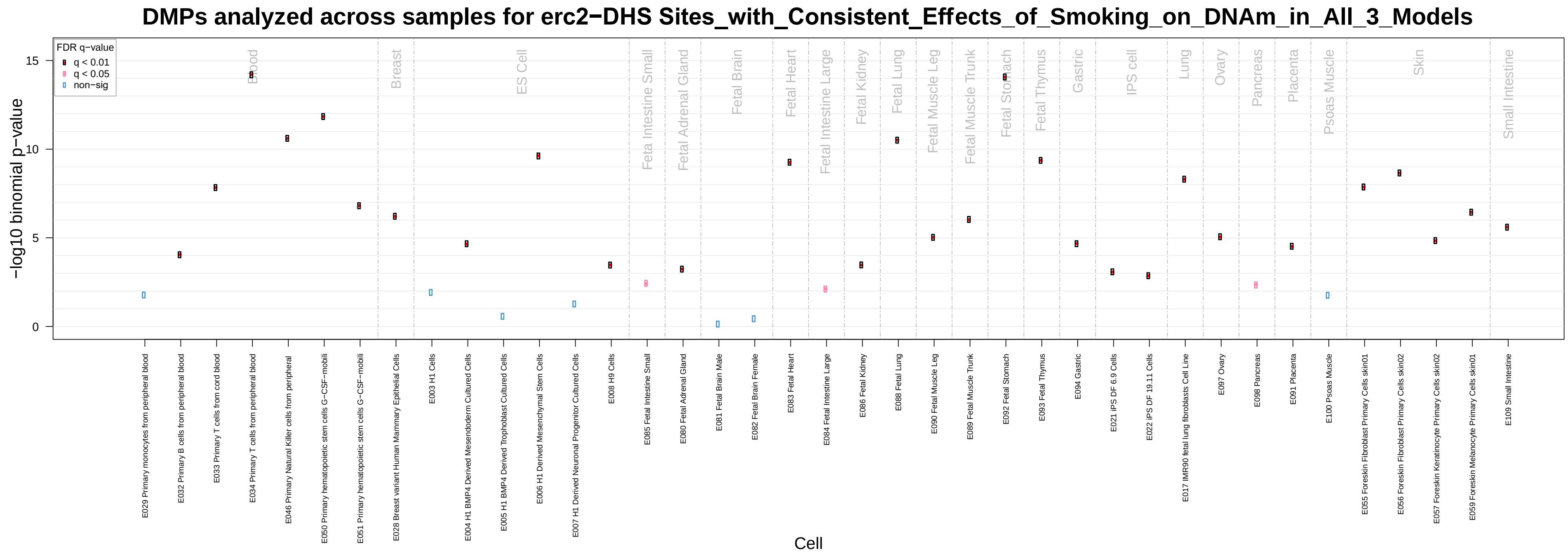

Note. Please refer to **Supplementary Table S9** for the corresponding data.

Figure S15

64 CpGs with potential DNAm → Current Smoking effects, based on consistent, nominally significant estimates across models

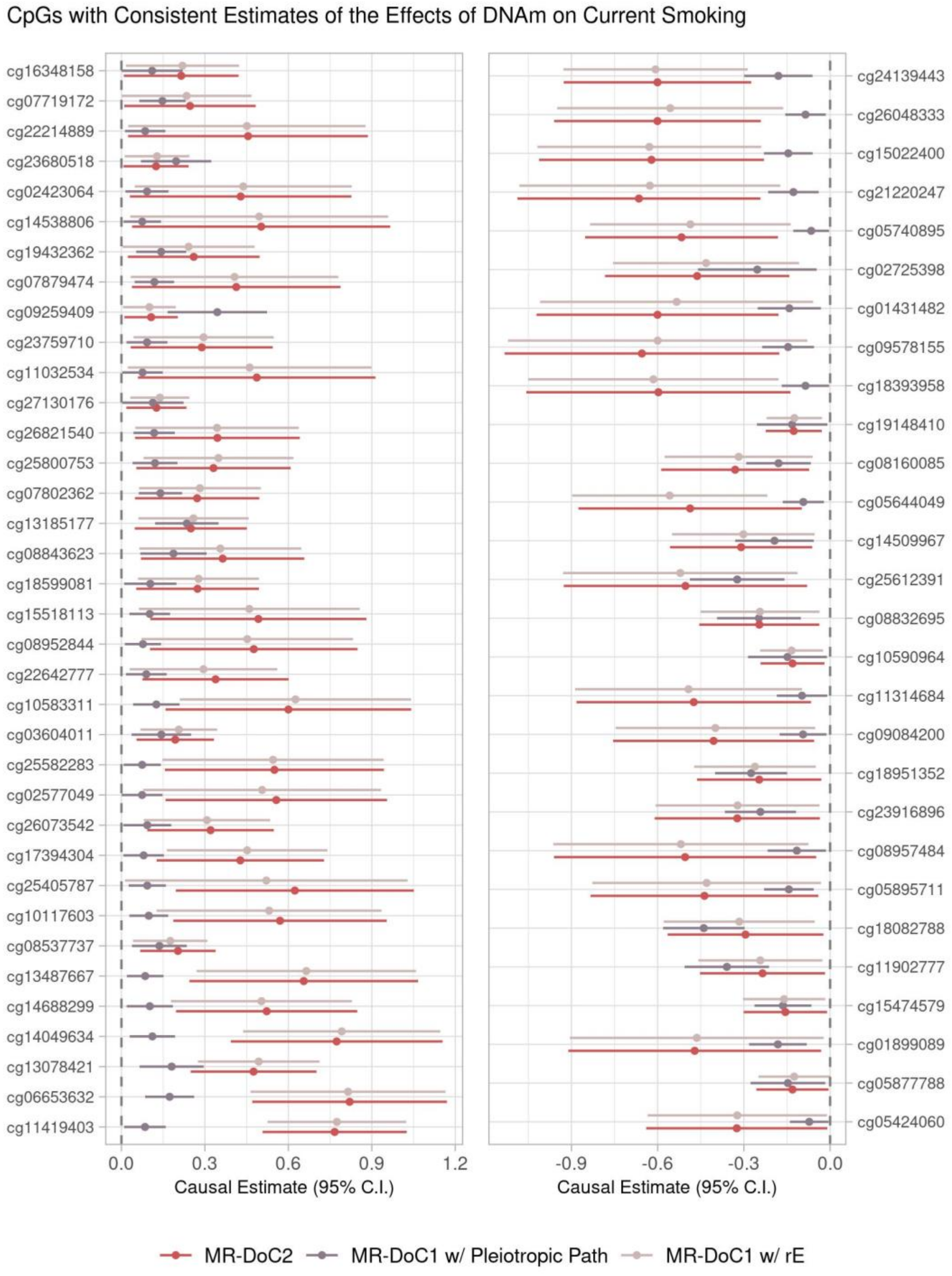

Note. These CpGs were used for the follow-up enrichment analyses with eFORGE[11] and Metascape[12]. None of these sites are in the MHC region. Please refer to **Supplementary Table S3** for the corresponding data.

Figure S16

Top Ontology Clusters in Metascape's Gene Annotation and Functional Enrichment Analyses of the 64 CpGs with Potential DNAm → Current Smoking effects

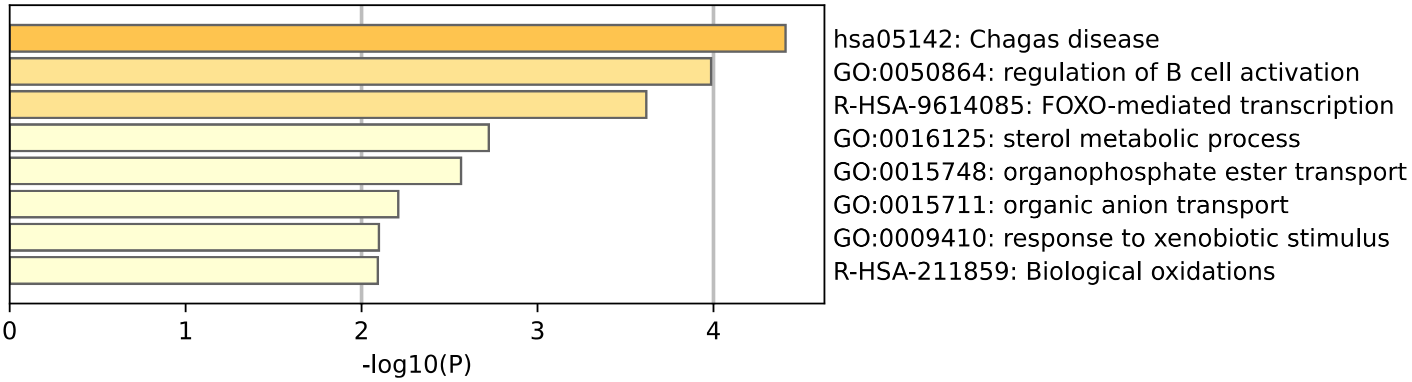

Note. The "NearestGene" IDs from *Supplementary Table S3* were used as the input data for Metascape[12]. None of the ontology terms were significant after multiple-testing correction. Please refer to **Supplementary Tables S10 and S11** for all annotation and enrichment results.

Figure S17

Enrichment Results for Gene-Ontology (GO) Processes in Metascape's Gene Annotation and Functional Enrichment Analyses of the 64 CpGs with Potential DNAm → Current Smoking effects

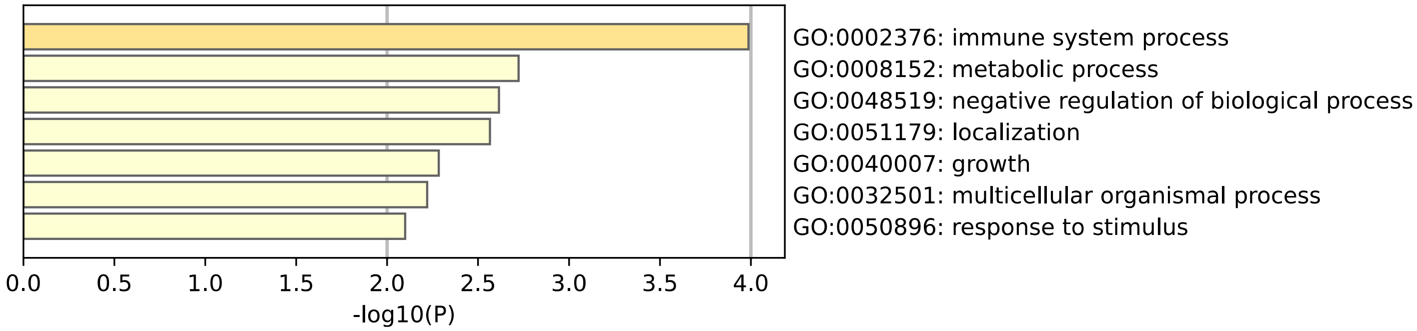

Note. The "NearestGene" IDs from *Supplementary Table S3* were used as the input data for Metascape[12]. None of the ontology terms were significant after multiple-testing correction. Please refer to **Supplementary Table S11** for all enrichment results.

Figure S18

eFORGE analyses of overlap between gene-regulatory chromatin states and the 64 CpGs with potential DNAm → Current Smoking effects

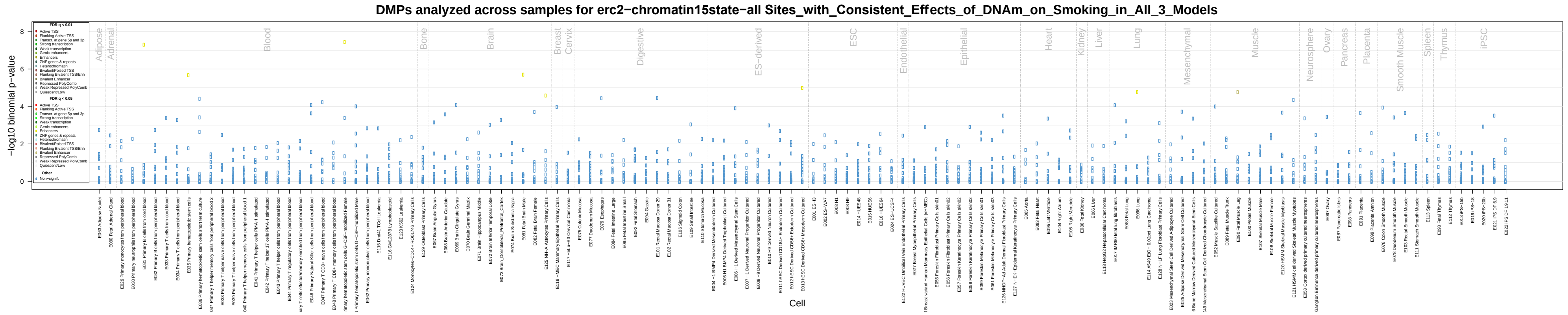

Note. Please refer to **Supplementary Table S12** for the corresponding data.

Figure S19

eFORGE analyses of overlap between histone-mark modifications *and the 64 CpGs with potential DNAm → Current Smoking effects*

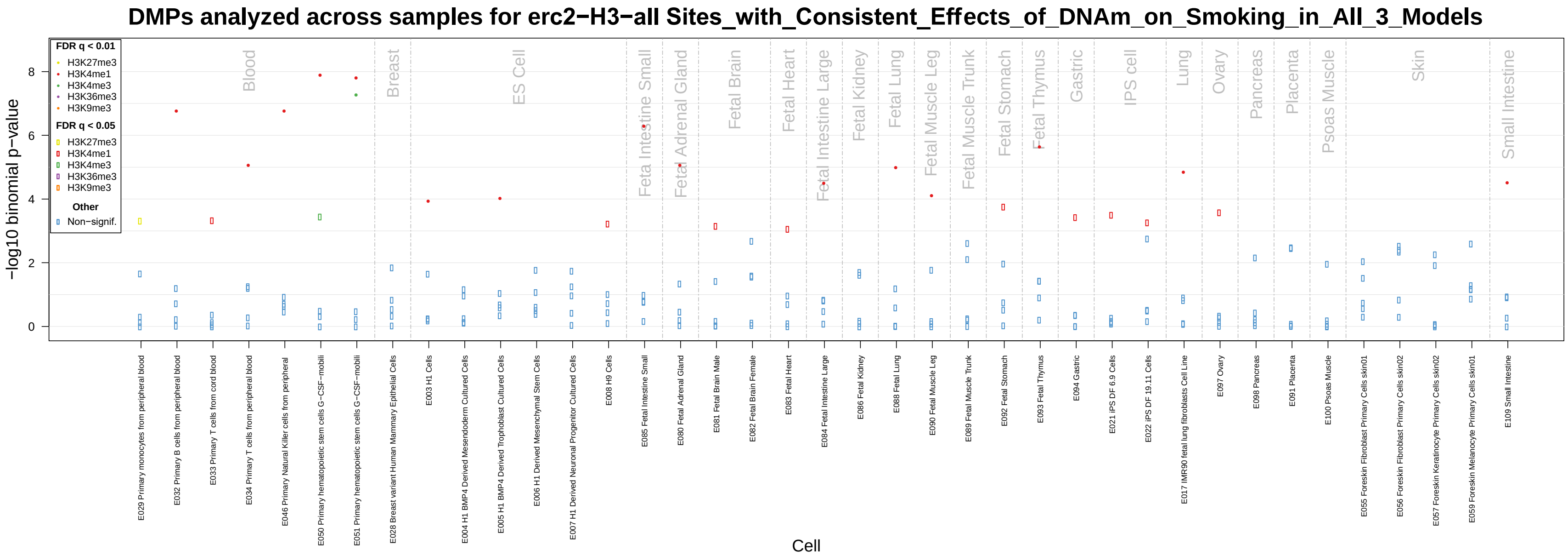

Note. Please refer to **Supplementary Table S13** for the corresponding data.

Figure S20

eFORGE analyses of overlap between DNase hypersensitivity (DHS) sites and the 64 CpGs with potential DNAm → Current Smoking effects

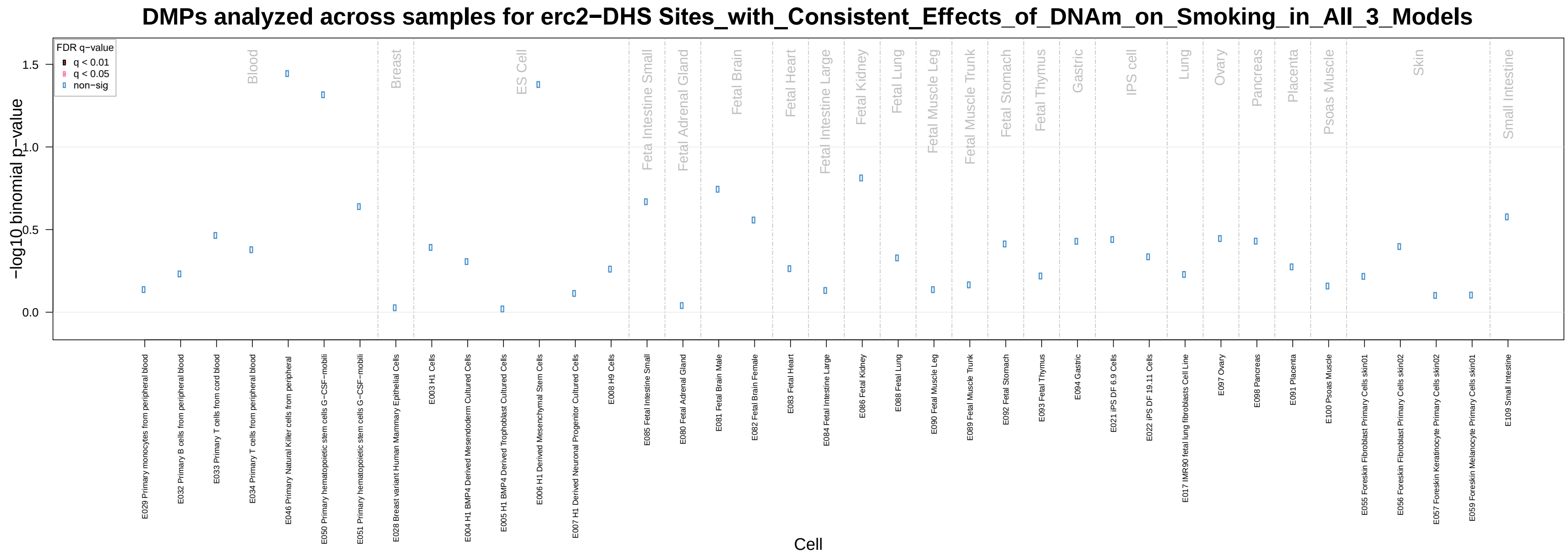

Note. Please refer to **Supplementary Table S14** for the corresponding data.

Figure S21

Follow-up eFORGE analyses of overlap between gene-regulatory chromatin states and the 21 CpGs enriched for overlap with Enhancers in the "Fetal Brain Male" sample in Figure S10/Table S5

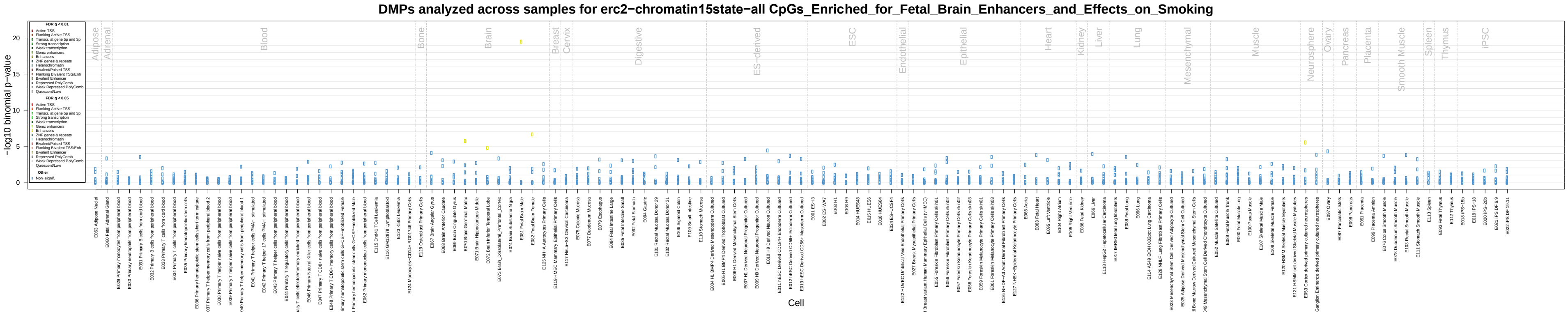

Note. Please refer to **Supplementary Table S15** for the corresponding data.

Figure S22

Follow-up eFORGE analyses of overlap between histone-mark modifications *and the 21 CpGs enriched for overlap with Enhancers in the "Fetal Brain Male" sample in Figure S10/Table S5*

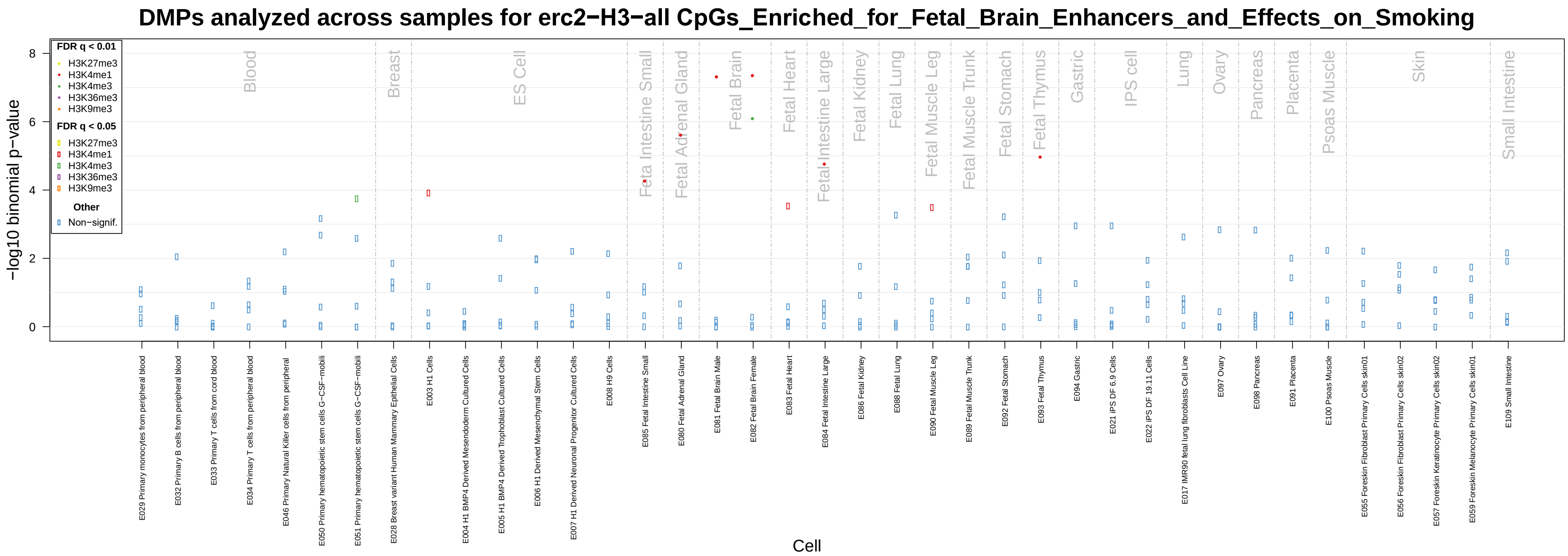

Note. Please refer to **Supplementary Table S16** for the corresponding data.

Figure S23

Follow-up eFORGE analyses of overlap between DNase hypersensitivity (DHS) sites and the 21 CpGs enriched for overlap with Enhancers in the "Fetal Brain Male" sample in Figure S10/Table S5

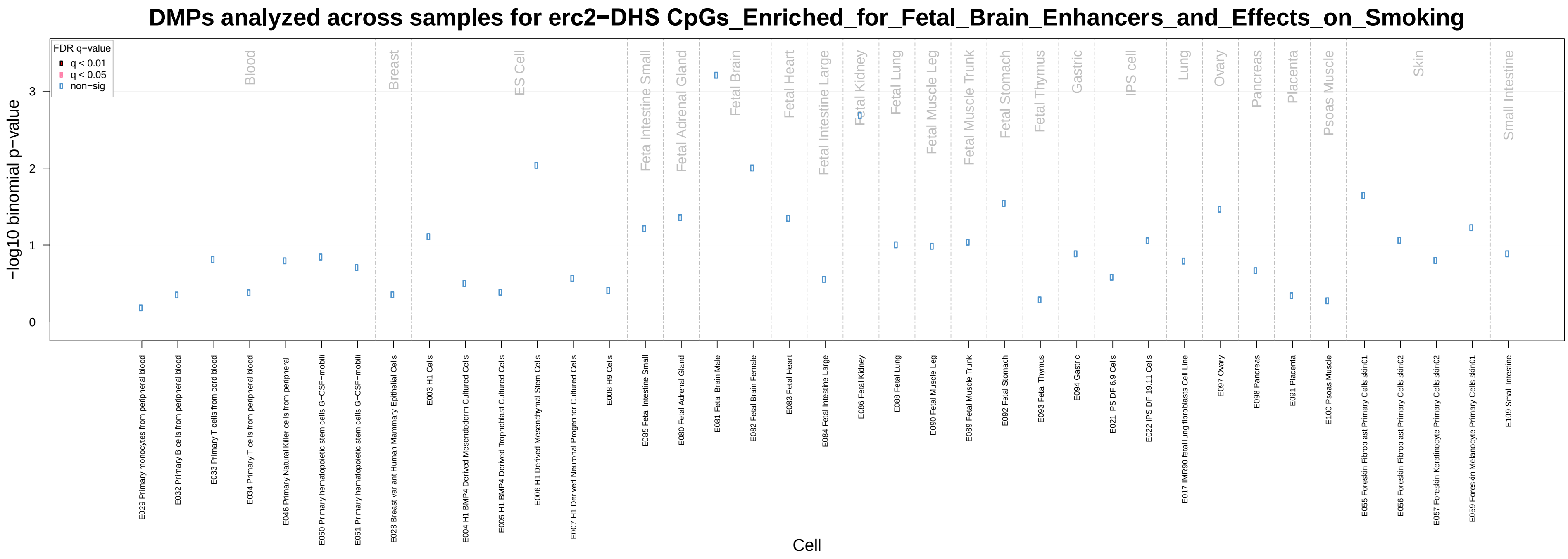

Note. Please refer to **Supplementary Table S17** for the corresponding data.

Figure S24

Follow-up eFORGE analyses of overlap between DNase hypersensitivity (DHS) sites and the 17 CpGs enriched for overlap with H3K4me3 modifications in the "Fetal Brain Female" samples in Supplementary Figure S14/Table S9

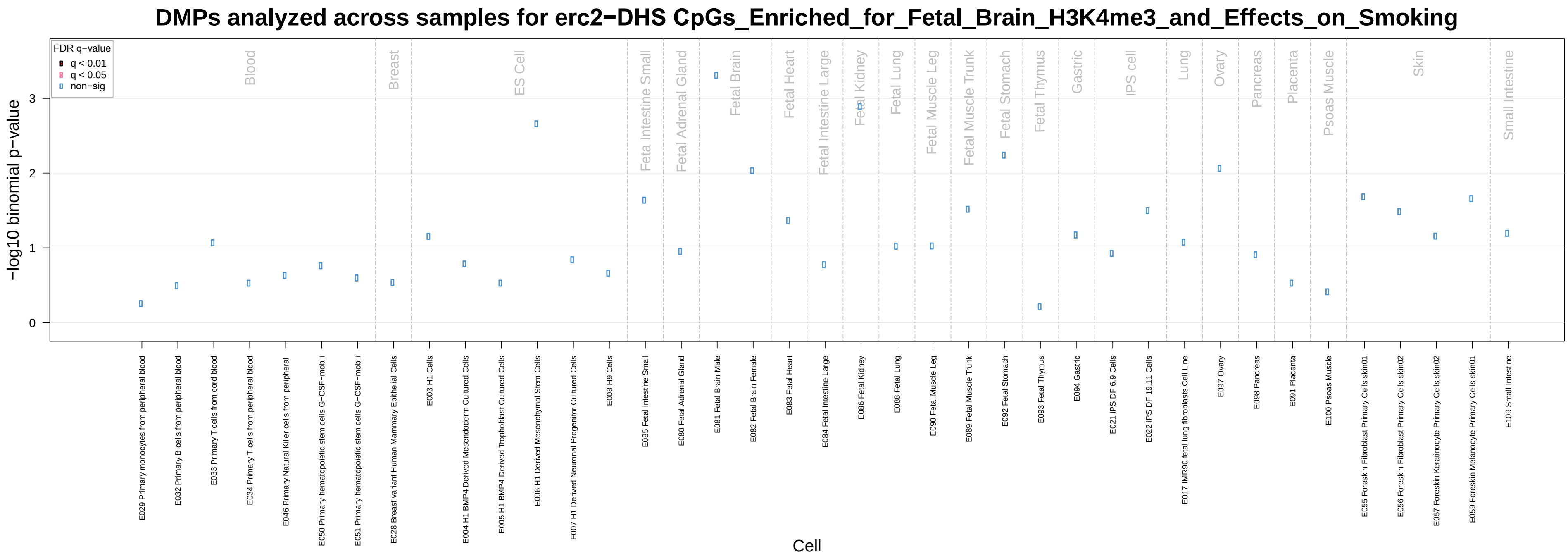

Note. Please refer to **Supplementary Table S20** for the corresponding data.

Figure S25

Estimated DNAm → Current Smoking effects at the 17 CpGs showing highly specific enrichment for overlap with gene-regulatory elements in the brain in Figure 7

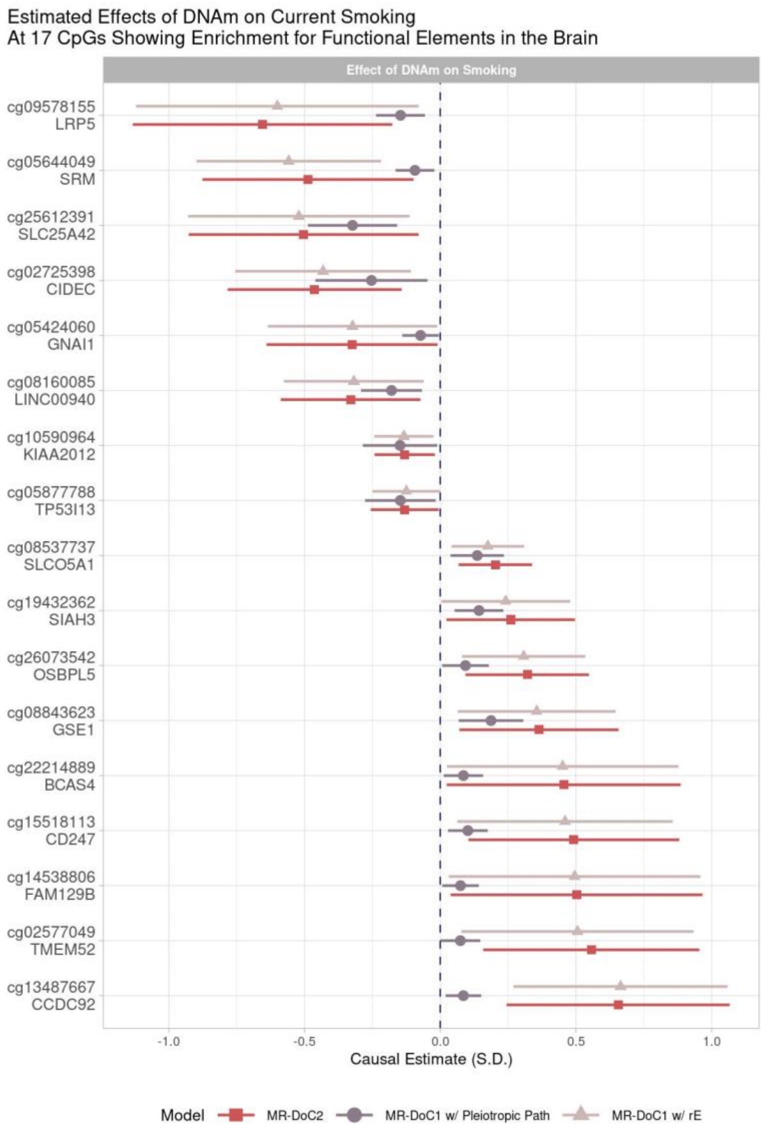

Note. The Y-axis shows the probe ID and the “Nearest Gene”. For the corresponding data, please refer to **Supplementary Table S3**.

Figure S26

eFORGE analyses of overlap between gene-regulatory chromatin states and the 18 CpGs underlying the enriched overlap with Enhancers in the "Lung" sample in Figure S10/Table S5

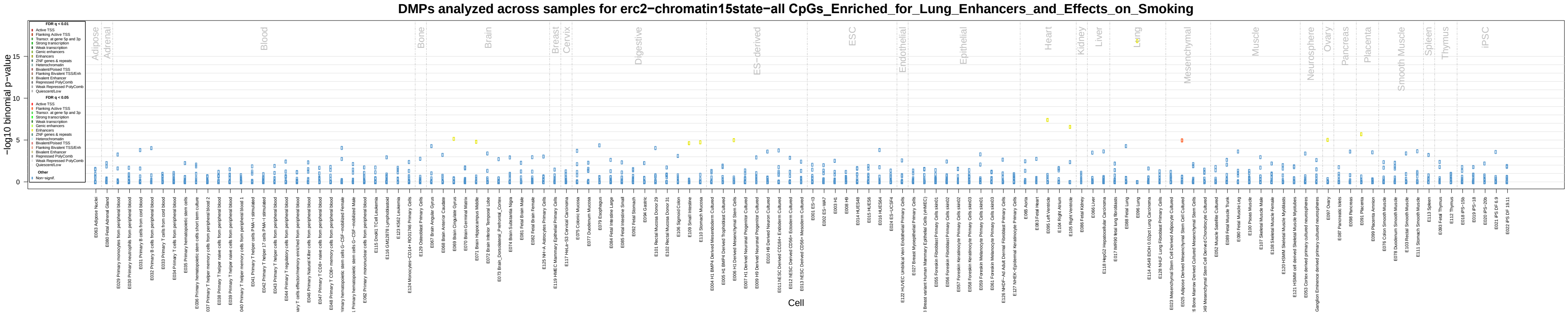

Note. Please refer to **Supplementary Table S21** for the corresponding data.

Figure S27

eFORGE analyses of overlap between histone-mark modifications and the 18 CpGs underlying the enriched overlap with Enhancers in the "Lung" sample in Figure S10/Table S5

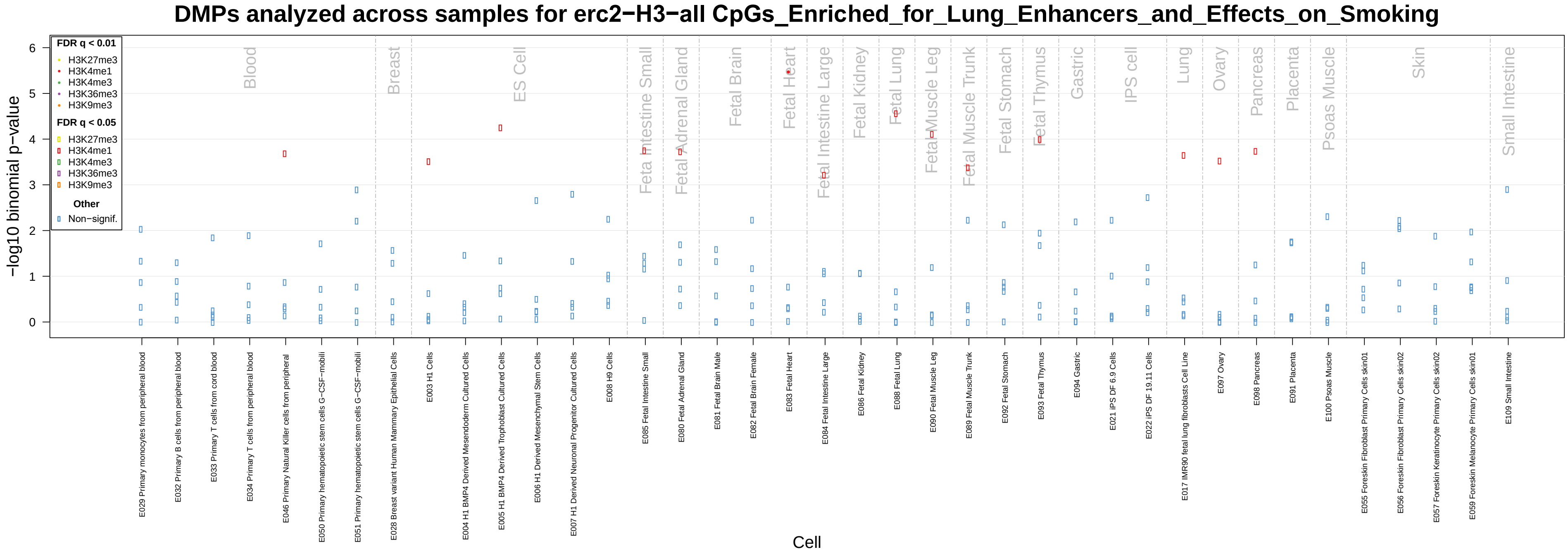

Note. Please refer to **Supplementary Table S22** for the corresponding data.

Figure S28

eFORGE analyses of overlap between DNase hypersensitivity (DHS) sites and the 18 CpGs underlying the enriched overlap with Enhancers in the "Lung" sample in Figure S10/Table S5

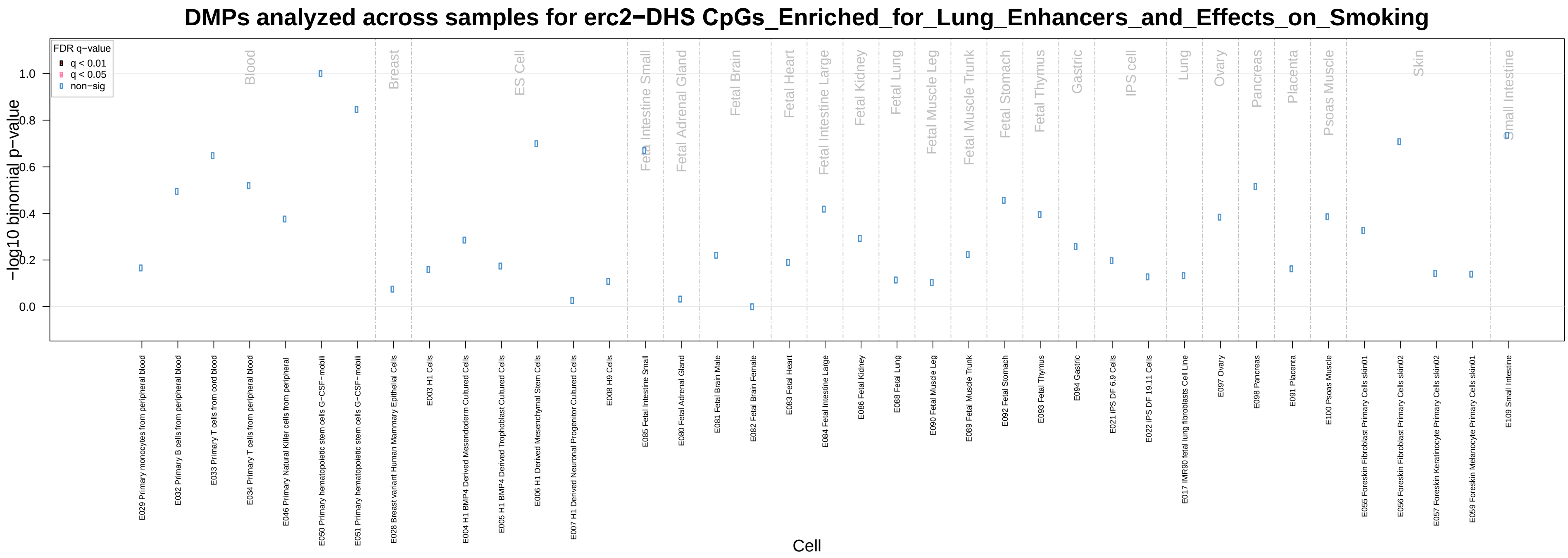

Note. Please refer to **Supplementary Table S23** for the corresponding data.

Figure S29

eFORGE analyses of overlap between gene-regulatory chromatin states and the 18 CpGs underlying the enriched overlap with Enhancers in the "Primary B cells from cord blood" sample in Figure S10/Table S5

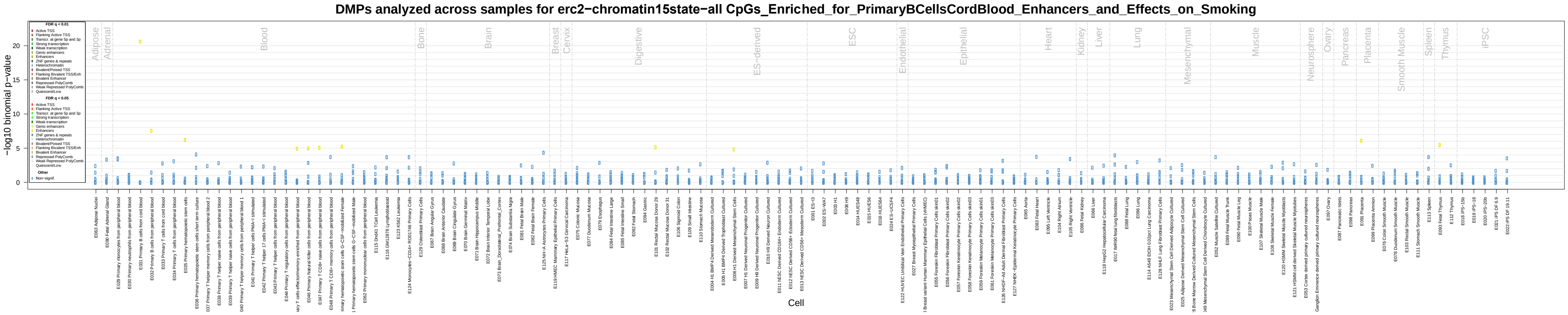

Note. Please refer to **Supplementary Table S24** for the corresponding data.

Figure S30

eFORGE analyses of overlap between histone-mark modifications and the 18 CpGs underlying the enriched overlap with Enhancers in the "Primary B cells from cord blood" sample in Figure S10/Table S5

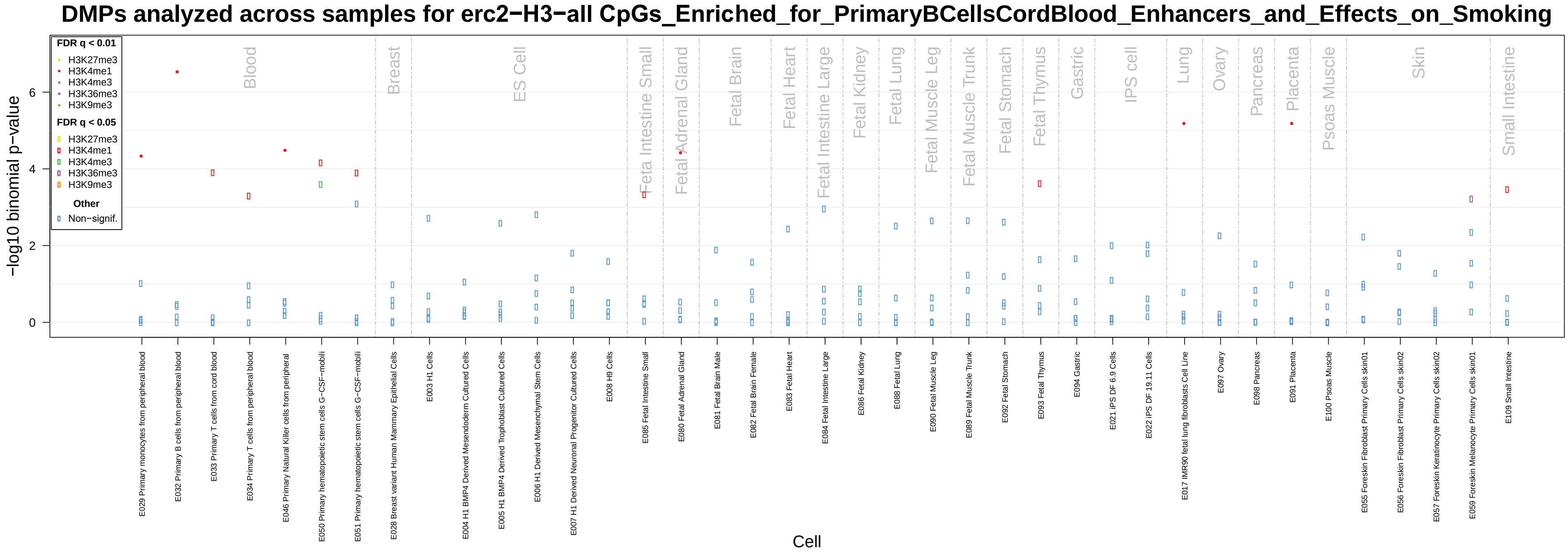

Note. Please refer to **Supplementary Table S25** for the corresponding data.

Figure S31

eFORGE analyses of overlap between DNase hypersensitivity (DHS) sites and the 18 CpGs underlying the enriched overlap with Enhancers in the "Primary B cells from cord blood" sample in Figure S10/Table S5

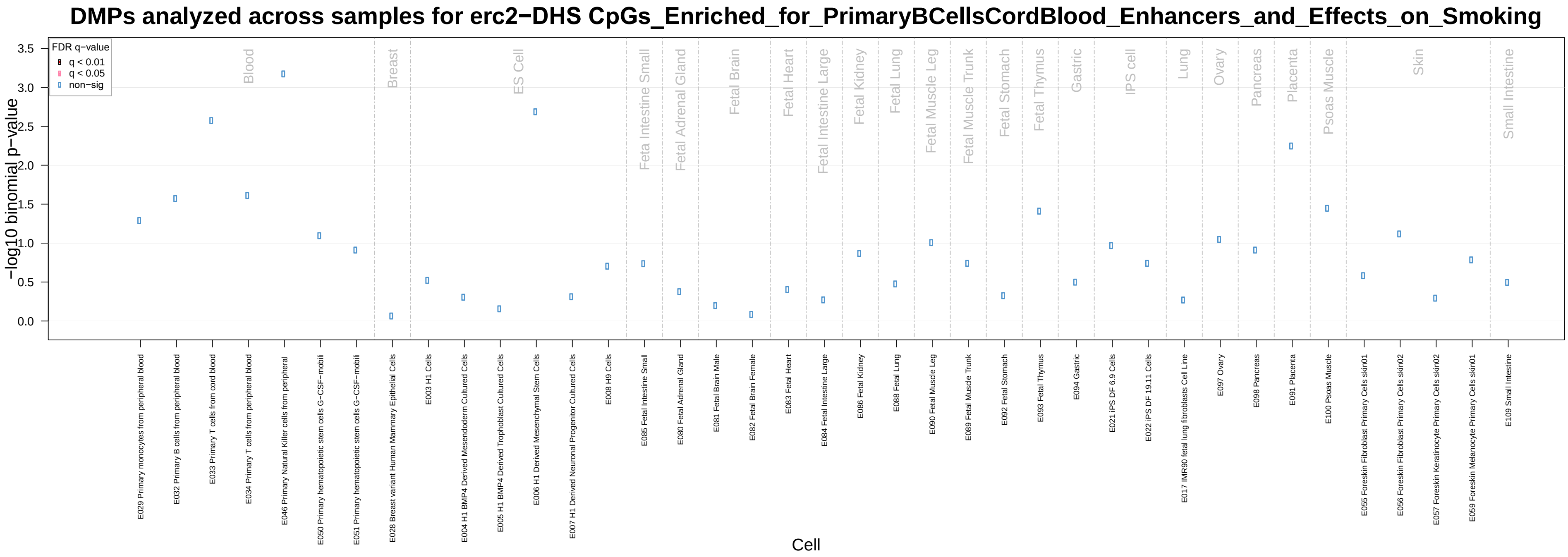

Note. Please refer to **Supplementary Table S26** for the corresponding data.

Figure S32

Upset plot of the intersection of CpGs with statistically significant (FDR <0.05) Former Smoking → DNAm effects in each of the three MR-DoC models

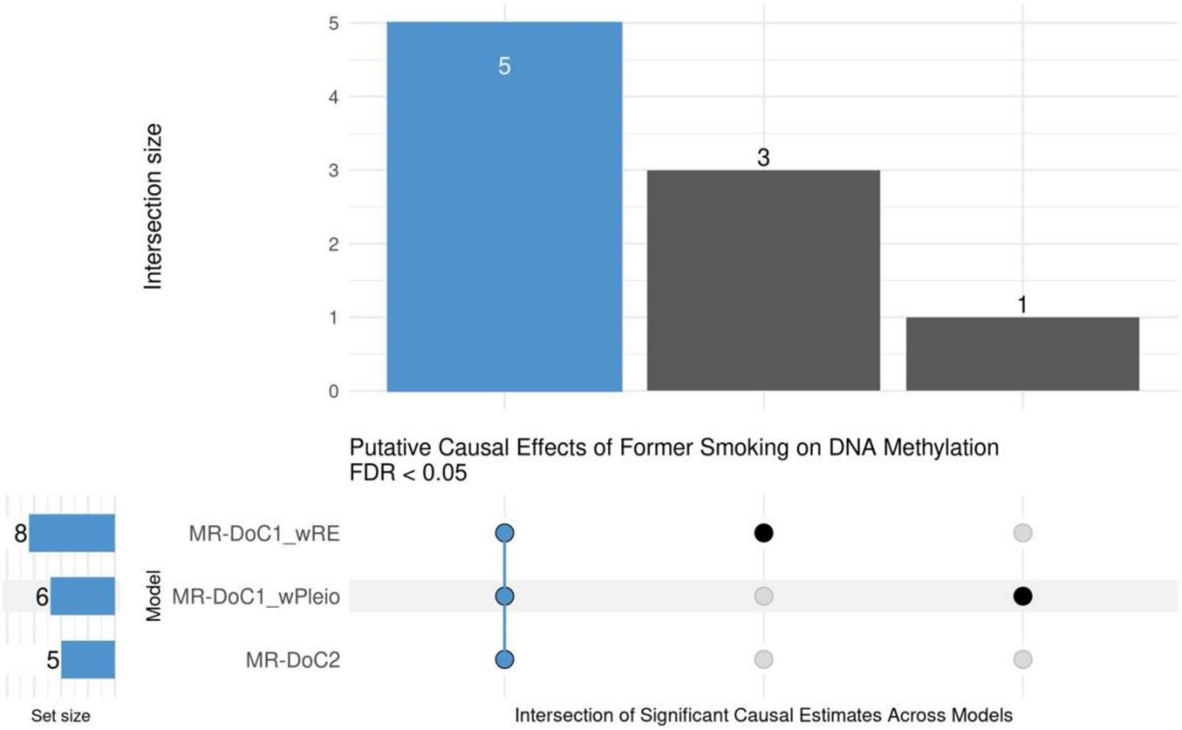

Note. Please refer to **Supplementary Table S27** for the corresponding data.

Figure S33

Upset plot of the intersection of CpGs with statistically significant (FDR <0.05) DNAm → Former Smoking effects in each of the three MR-DoC models

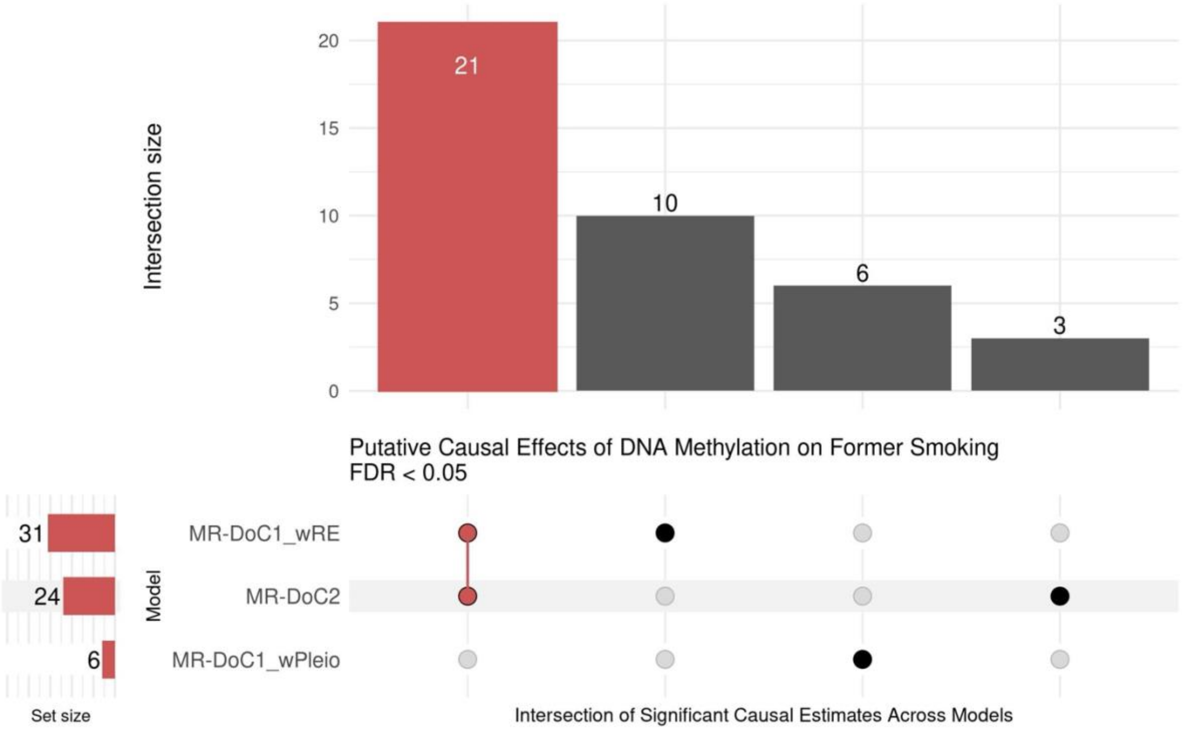

Note. Please refer to **Supplementary Table S29** for the corresponding data.

Figure S34

Estimated DNAm → Former Smoking effects at the two former-smoking-associated CpGs that showed robust evidence of DNAm → Current Smoking effects

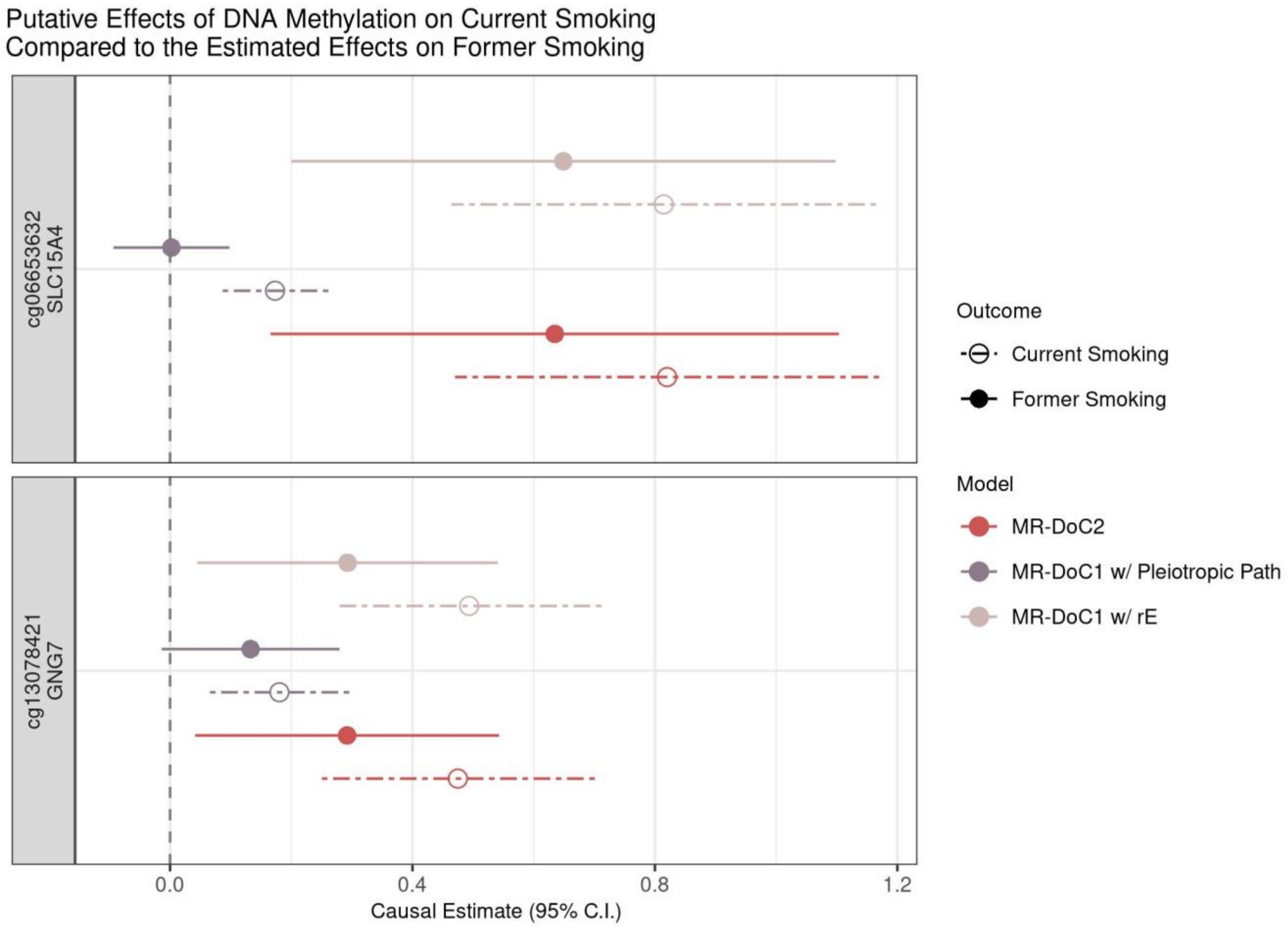

Note. Please refer to **Supplementary Tables S3 and S29** for the corresponding data.

Figure S35

Prior EWAS association statistics of smoking-associated CpGs stratified by whether the CpG was identified as having an mQTL allelic score with F-statistic >10 in the current study

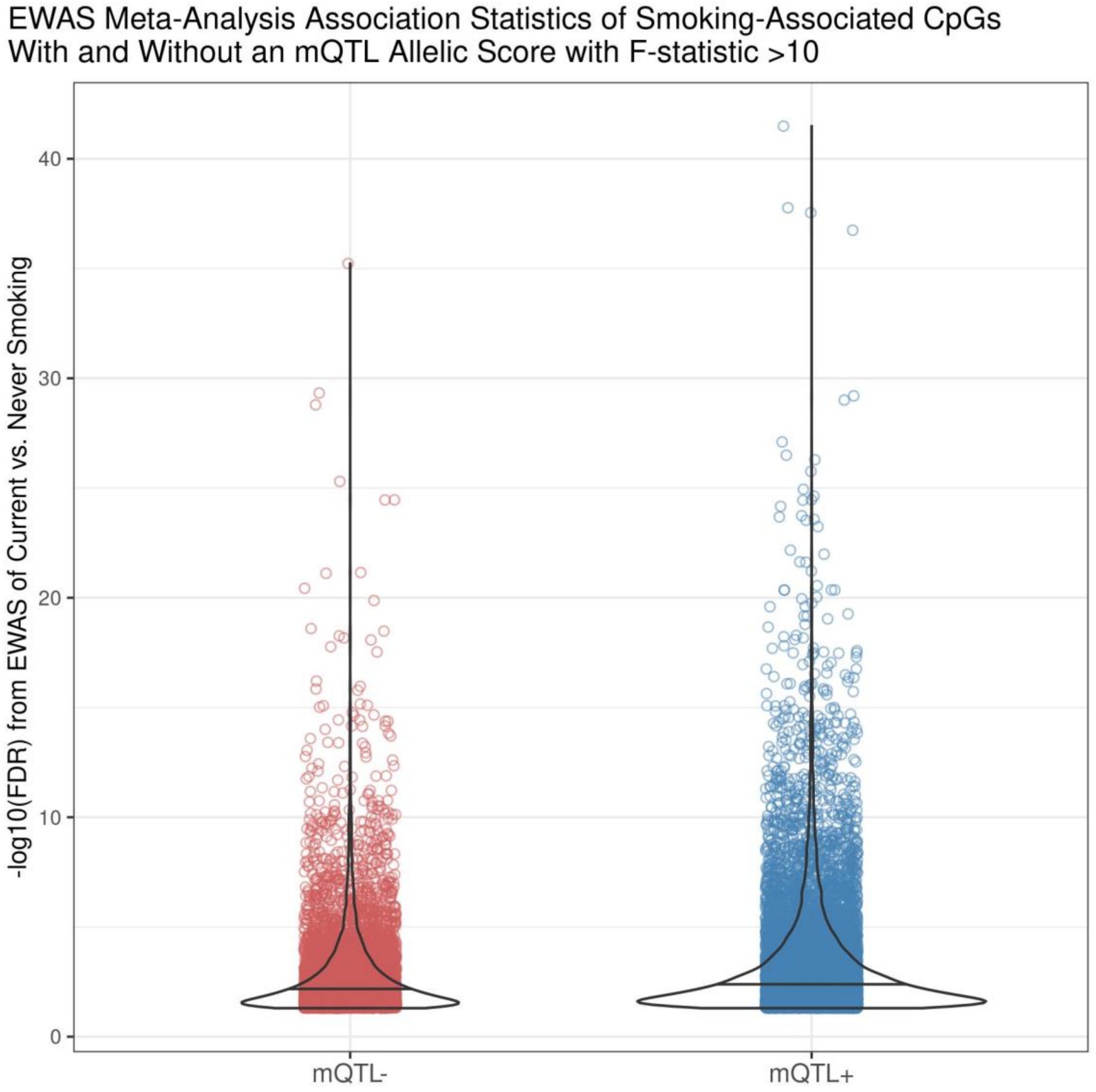

On the X-axis, "mQTL-" indicates the CpGs without an mQTL allelic score with F >10 (5,816 CpGs), and "mQTL+" indicates the CpGs with an mQTL allelic score with F >10 (11,124 CpGs). The Y-axis shows the -log10(FDR) values of the association results from the previous EWAS meta-analysis of current vs. never smoking[13]. The "mQTL-" CpGs were not tested for DNAm → Smoking causal effects in the current study.

References

1. Ligthart, L. *et al.* The Netherlands Twin Register: Longitudinal Research Based on Twin and Twin-Family Designs. *Twin Research and Human Genetics* **22**, 623–636 (2019).
2. Minică, C. C., Dolan, C. V., Boomsma, D. I., De Geus, E. & Neale, M. C. Extending Causality Tests with Genetic Instruments: An Integration of Mendelian Randomization with the Classical Twin Design. *Behavior Genetics* **48**, 337–349 (2018).
3. Castro-de-Araujo, L. F. S. *et al.* MR-DoC2: Bidirectional Causal Modeling with Instrumental Variables and Data from Relatives. *Behavior Genetics* **53**, 63–73 (2023).
4. Singh, M. *et al.* Using Instrumental Variables to Measure Causation over Time in Cross-Lagged Panel Models. *Multivariate Behavioral Research* **59**, 342–370 (2024).
5. Auton, A. *et al.* A global reference for human genetic variation. *Nature* **526**, 68–74 (2015).
6. Haplotype Reference Consortium. A reference panel of 64,976 haplotypes for genotype imputation. *Nature Genetics* **48**, 1279–1283 (2016).
7. Francioli, L. C. *et al.* Whole-genome sequence variation, population structure and demographic history of the Dutch population. *Nature Genetics* **46**, 818–825 (2014).
8. Price, A. L. *et al.* Principal components analysis corrects for stratification in genome-wide association studies. *Nature Genetics* **38**, 904–909 (2006).
9. Saunders, G. R. B. *et al.* Genetic diversity fuels gene discovery for tobacco and alcohol use. *Nature* **612**, 720–724 (2022).
10. Vilhjálmsson, J. *et al.* Modeling Linkage Disequilibrium Increases Accuracy of Polygenic Risk Scores. *The American Journal of Human Genetics* **97**, 576–592 (2015).
11. Breeze, C. E. *et al.* eFORGE: A Tool for Identifying Cell Type-Specific Signal in Epigenomic Data. *Cell Reports* **17**, 2137–2150 (2016).
12. Zhou, Y. *et al.* Metascape provides a biologist-oriented resource for the analysis of systems-level datasets. *Nature Communications* **10**, (2019).
13. Joehanes, R. *et al.* Epigenetic Signatures of Cigarette Smoking. *Circulation. Cardiovascular genetics* **9**, 436–447 (2016).
